# The RCAR12-CAP1-OST1 module controls ABA-mediated stomatal closure in Arabidopsis

**DOI:** 10.1371/journal.pgen.1012092

**Published:** 2026-04-28

**Authors:** Xiaoyi Li, Qian Zhang, Jiangjie Kang, Lianxin Jiang, Yihan Feng, Juan Du, Chaowen Xiao, Jianglan Wei, Qirong Wen, Ying Wang, Jianmei Wang, Yi Yang

**Affiliations:** 1 Key Laboratory of Bio-Resources and Eco-Environment of Ministry of Education, College of Life Sciences, Sichuan University, Chengdu, China; 2 Chengdu Botanical Garden-Sichuan University Joint Laboratory for Ex Situ Conservation and Resource Utilization of Mountain Plants, Chengdu, China; 3 Southwest Bio-resources R&D Key Laboratory of Sichuan Province, Sichuan University, Chengdu, China; Harvard Medical School, UNITED STATES OF AMERICA

## Abstract

Phytohormone abscisic acid (ABA) induces the stomatal closure in plants under drought stress, a process requiring actin reorganization. Yet how ABA perception couples with cytoskeletal dynamics in stomatal closure remains unclear. In this study, we report that Arabidopsis cyclase-associated protein 1 (CAP1) functions as a negative regulator of ABA-induced drought responses through modulating actin network organization in Arabidopsis. Further analyses demonstrate that CAP1 interacts with RCAR12 suppresses actin filaments (F-actin) disassembly, whereas ABA disrupts the CAP1-RCAR12 interaction, thus recovering CAP1’s depolymerization activity. Further, ABA-activated OST1 (OPEN STOMATA 1) phosphorylates CAP1 in Arabidopsis. OST1-mediated phosphorylation of CAP1 attenuates CAP1 binding to ADF4 and inhibits the ADF4-CAP1 complex. This inhibition leads to F-actin stabilization, which in turn maintains stomata in a closed state. This study demonstrates that CAP1 orchestrates ABA-induced stomatal closure under drought stress in Arabidopsis. Specifically, CAP1 acts by coupling ABA signaling to dynamic reorganization of actin cytoskeleton.

## Author summary

It is well established that the phytohormone abscisic acid (ABA) plays a crucial role in regulating stomatal closure. The cytoskeleton, an essential structural component of plant cells—including guard cells—forms a dynamic network that is integral to cellular signaling. Although actin filaments (F-actin) have been implicated in ABA-induced stomatal closure, the precise regulatory mechanisms remain largely unclear. In this study, we demonstrate that CAP1 (Cyclase-Associated Protein 1) interacts with ABA receptors RCARs (Regulatory Components of ABA Receptors), and this interaction inhibits CAP1’s ability to depolymerize actin filaments. This repression is relieved upon ABA binding to its receptors. Furthermore, we show that CAP1 is phosphorylated by the protein kinase OST1 (OPEN STOMATA 1). Phosphorylation of CAP1 attenuates its interaction with ADF4 (Actin Depolymerizing Factor 4), leading to F-actin stabilization and sustained stomatal closure. Together, these findings reveal a molecular framework that couples ABA signaling to actin cytoskeleton remodeling during drought stress in plants.

## Introduction

Limited water availability is one of the challenges for plant survival and reproduction. To overcome water scarcity, plants specialize their epidermal cells into dynamically adjustable stomata on leaves, which optimize CO_2_ uptake against transpirational water loss [1,2]. In this progress, phytohormone ABA (abscisic acid) is known as a critical regulator of stomatal closure. ABA accumulates under drought stress, subsequently conveys nuclear and turgor signals by regulating complexes formed between receptors PYR/PYL/RCAR proteins (hereafter referred to as RCARs), PP2Cs (Protein Phosphatases Type 2c) and OPEN STOMATA 1 (OST1, also known as SnRK2.6, one of SnRK2s) [3–5]. As a long-term strategy for survival, ABA-activated SnRK2s phosphorylate the ABA-responsive transcriptional factors, including ABI5 (ABA Insensitive 5) and ABFs (ABA-Responsive Element Binding Factors), to induce the expression of stress-responsive genes [6,7]. On the other hand, as a short-term response to drought stress, the activation of H+, K+, and anion channel proteins reduce turgor pressure in guard cells and then triggers stomatal closure [8–11].

Accumulated evidence underscores the importance of actin cytoskeleton reorganization-actin disassembly and reassembly-for stomatal closure, as pharmacological disruption of this process impairs the response [12–15]. For instance, ABA-induced stomatal closure is inhibited by Jasp (Jasplakinolide, an actin-stabilizing agent) but triggered by LatB (Latrunculin B, an actin cytoskeleton disruptor) [16]. Live-cell imaging reveals that the cortical actin array during stomatal closure undergoes disassembly and reassembly, shifts from a radial array to a randomly organized network, and subsequently to a longitudinal array [17]. Genetic disruption of a plethora of ABPs (Actin-Binding Proteins) affects the stomatal closure. Mutations in ARP2/3 (Actin-Related Proteins 2/3), SCAR/WAVE (WASP Family Verprolin Homologous Protein/Suppressor of cAMP Repressor) complex, and ADF (Actin-Depolymerizing Factor) reduce dark- or ABA-induced stomatal closure [13,15,18,19]. These findings demonstrate the importance of actin reorganization in stomatal movement.

Actin dynamic is precisely regulated by ABPs, while their activities are further regulated by upstream signaling messengers including ABA, Ca^2^+, phosphoinositide lipids, pH and post-translational modifications [20,21]. Recent researches show that the formation of ABA-PYR/PYL/RCARs-PP2C complex releases CKL2 (Casein Kinase 1-Like Protein 2), which subsequently inhibits the activity of ADF4 and then mediates the redirection and repolymerization of F-actin in the final process of stomatal closure [15,22]. PI3P (PI 3-phosphate) binds to SCAB1 (Stomatal Closure-Related Actin-Binding Protein1) and inhibits its ability to bundle F-actin, leading to F-actin destabilization and vesicle shrinkage at the onset of stomatal closure [23]. However, the molecular mechanisms of the stimulus-response coupling pathway mediated by the actin cytoskeleton in guard cells remain unknown.

Previous study indicates that CAP1 (Cyclase-associated Protein 1) interacts with the ABA receptor RCAR8 [24], suggesting a link between ABA signaling and the actin cytoskeleton. CAP is a novel key regulator of actin dynamics and spatiotemporally controls assembly and disassembly of F-actin. N-terminal domain of CAP promotes F-actin disassembly by enhancing ADF-mediated actin severing and depolymerization, while the central and C-terminal domains remove ADF from ADP-G-actin and catalyze nucleotide exchange on G-actin [25–28]. ADF is a conserved actin disassembly regulator by promoting F-actin depolymerization and severing [29,30]. The disassembly activity of the CAP-ADF complex regulates the size and distribution of the monomeric actin pool, which controls the growth rate and orientation of F-actin [31]. As mentioned above, the process of ABA-mediated stomatal closure is accompanied by reassembly and disassembly of F-actin. Thus, the activity of CAP1-ADF may also be tightly regulated during stomatal closure.

In this study, our findings demonstrate that the F-actin disassembly function of CAP1 is inhibited upon binding to the ABA receptor RCAR12. Further analysis reveals that the formation of the ABA-RCAR12 complex releases CAP1 and increased the activity of the CAP1-ADF4 complex on disassembling F-actin. In addition, OST1, a core component of ABA signaling, phosphorylates CAP1 to suppress its binding to ADF4, thus inhibiting F-actin reassembly and maintaining stomatal closure during drought stress. Thus, our study establishes a direct link between ABA and actin cytoskeleton, demonstrating that ABA-induced actin reorganization in guard cells promotes stomatal closure under drought stress.

## Results

### Arabidopsis CAP1 mainly interacts with ABA receptors

**Fig 1:**
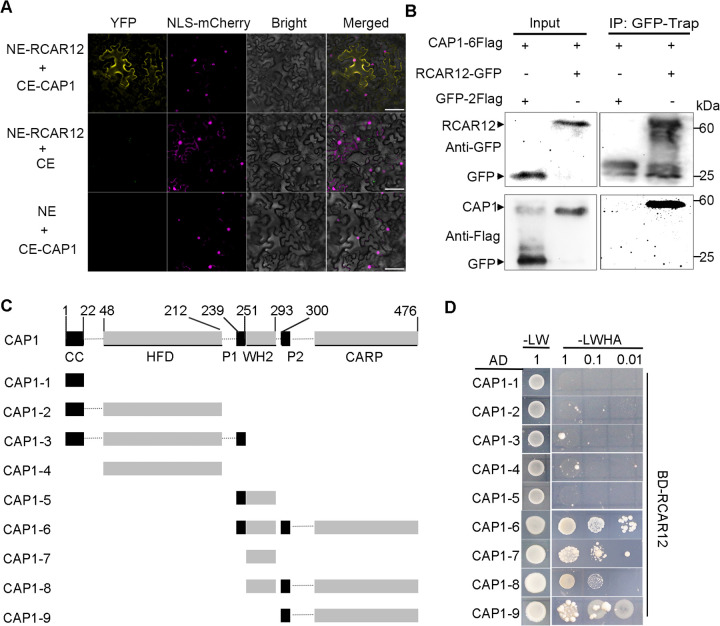
RCAR12 interacts with CAP1. (A) Bimolecular Fluorescence Complementation (BiFC) assay. The signal of fluorescence was detected in the leaf epidermal cells of *N. benthamiana* after 3 days cultivation by a fluorescence microscope. CE: *p*SPYCE vector; NE: *p*SPYNE vector; NLS-mCherry indicates nuclear localization. Scale bars = 50 μm. The images are the representative of three independent experiments (*n* = 3). (B) Co-IP results demonstrating the interaction of CAP1 with RCAR12 in the leaves of *N. benthamiana*. GFP-2Flag was set as a negative control. The images are the representative of two independent experiments (*n* = 2) with the same results. (C) Schematic domain organization of CAP1 and nine truncated CAP1 variants. CC, coiled coil domain; HFD, helically folded domain; P1 and P2, proline-rich region 1 and 2; WH2, Wiscott Aldrich syndrome protein-homology 2 domain; CARP, CAP-retinitis pigmentosa. (D) Yeast-two-hybrid interactions CAP1’s variants with RCAR12 by spotting serial dilutions (1:10, 1:100) of yeast on selective dropout media. The images are the representative of three independent experiments (*n* = 3).

Previous study reveals that the C-terminus of CAP1 interacts with ABA receptor RCAR8 [24]. To analyze whether the interaction between CAP1 and RCARs is structure-specific, we evaluated the interactions between CAP1 and all members of the RCARs family. Yeast two-hybrid (Y2H) assay showed that all of RCARs interacted with CAP1 in yeast cells (S1A Fig). The fluorescent bimolecular complementation (BiFC) assays indicated that CAP1 interacted with RCAR8 and all dimeric RCARs in tobacco cells, including RCAR11, RCAR12, RCAR13 and RCAR14 (Fig 1A and S1B Fig). No fluorescent signal was found for the other receptors with CAP1 (only RCAR3 is shown) and the negative combinations (Figs 1A and S1B Fig). GST-Pull down also showed the interactions between CAP1 and RCAR8, RCAR11, RCAR12, RCAR13 and RCAR14 (S1C Fig). Considering that RCAR8/11/12/14 are expressed in guard cells [32], the data from the ePlant browser (https://bar.utoronto.ca/eplant/) indicate that RCAR12 is enriched in leaves, which is consistent with the fluorescence of RCAR12-GFP is distributed in guard cells (S2 Fig). Thus, we selected RCAR12 for further analysis. The co-immunoprecipitation (Co-IP) assay confirmed that CAP1 interacted with RCAR12 in plant cells (Fig 1B).

To determine the domain in CAP1 responsible for the interaction with RCAR12, we fused nine truncated CAP1 variants to the prey vector *p*GADT7 (AD) and conducted Y2H assays (Fig 1C). RCAR12 interacted with CAP1–6, CAP1–7, CAP1–8 and CAP1–9 (Fig 1D), suggesting that WH2 (Wiscott Aldrich Syndrome Protein Homology 2) and CARP (CAP-retinitis Pigmentosa) domains of CAP1 are responsible for its interaction with RCAR12. However, RCAR12 did not associate with CAP1–5, which contains WH2 and P1 domains. To test the oligomerization of CAP1, the nine truncated CAP1 variants were fused to the bait vector *p*GBKT7 (BD). Results showed that the C-terminal domain of CAP1 (CAP1–6) was capable of interacting with the intact N-terminal domain (CAP1–2), with itself, or with full-length CAP1 in yeast, as shown in S3A Fig. These interactions suggest that CAP1 can form oligomerization. The interactions between full-length CAP1 and truncated CAP1 in yeast cells further reveal the ubiquitous oligomerization and self-inhibition of CAP1 domains (S3A Fig). CAP1–5 showed no interaction with several variants (e.g., CAP1–1/2/3/4/7; S3A Fig), among other combinations that were omitted from the figure. In addition, we also confirmed the CAP1 display self-association in plant cells (S3B Fig).

### CAP1 plays a negative role in response to drought stress in Arabidopsis

**Fig 2:**
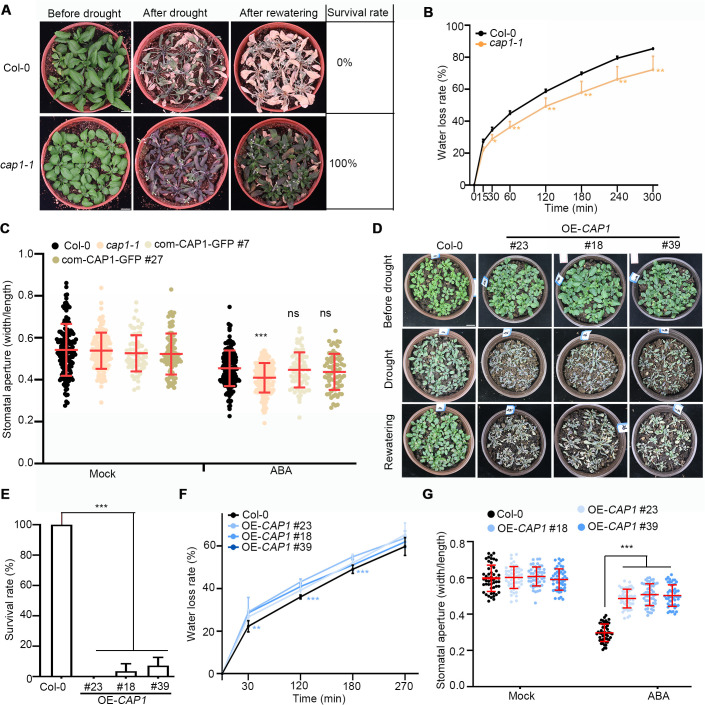
*CAP1* negatively regulates ABA-induced stomatal closure. (A) The *cap1-1* mutant showed enhanced drought tolerance compared to the wild-type plants Col-0. Three-week-old seedlings were subjected to drought stress by withholding water for 15-day and then rewatered for recovery. Over ten plants per genotype were analyzed for survival rates. The images are the representative of three independent experiments (*n* = 3) with similar results. Scale bars = 1 cm. (B) The analysis of cumulative leaf transpirational water loss in Col-0 and *cap1-1* plants. Six leaves were analyzed per genotype. Values are mean ± SD (*n* = 3). The experiment was repeated three times. *P < 0.05, **P < 0.01 determined by Student’s *t*-test. (C) ABA-induced stomatal closure in Col-0, *cap1-1* and complementation lines (com-CAP1-GFP) plants. Mock and ABA: without and with the treatment of 20 μM ABA for 1 h. The stomatal aperture was measured using ImageJ software (*n* ≥ 50). ***P < 0.001 determined by one-way ANOVA with Dunnett’s test. ns indicates no significant difference. (D) Over-expressing *CAP1* (OE-*CAP1*) plants reduced drought resilience compared to the Col-0 plants. Two-week-old were subjected to drought stress by withholding water for 10-day and then rewatered for recovery. The images are the representative of four independent experiments (*n* = 4) with similar results. Scale bar = 1 cm. (E) The analysis of seedlings survival rates of plants in (D). Over ten plants per genotype were analyzed for survival rates. Values are mean ± SD (*n* = 3). ***P < 0.001 determined by one-way ANOVA with Dunnett’s test. (F) Water loss of Col-0 and OE-*CAP1* plant leaves. Six leaves were analyzed per genotype. Values are mean ± SD (*n* = 3). **P < 0.01, ***P < 0.001 determined by Student’s *t*-test. The experiment was repeated three times with similar results. (G) Quantitative analysis of ABA-induced stomatal closure in Col-0 and OE-*CAP1* plants. Two or three-week-old leaves were treated with or without 20 μM ABA for 1 h. The stomatal aperture was measured using ImageJ software. Values are mean ± SD (*n* ≥ 50). ***P < 0.001 determined by one-way ANOVA with Dunnett’s test. Three biological experiments were performed with similar results.

To determine the functions of CAP1 in ABA signaling, we generated *CAP1-GFP* transgenic plants under the native promoter (com-CAP1-GFP; S4A Fig and S4B Fig). Given that GFP signals demonstrate CAP1 expression in guard cells (S4C Fig), we hypothesized that CAP1 plays a role in stomatal movement. To test this notion, we obtained the *cap1–1* mutant (SALK_112802) from the ABRC stock center. In drought tolerance assay, the *cap1–1* mutants exhibited purple leaves and fully recovered after rewatering showing a 100% survival rate. In contrast, after 15 days of water withholding, all Col-0 plants had died (Fig 2A). In water loss assay, detached leaves from *cap1–1* mutants exhibited a slower rate of water loss compared to those from Col-0 plants (Fig 2B). The assessment of the effect of ABA on stomatal movement revealed that stomatal apertures (width/length) of the *cap1–1* leaves had smaller stomatal apertures compared to those of Col-0 plants treated with ABA, while no significant difference under normal conditions (Fig 2C and S5A Fig). We verified the phenotype of ABA-induced stomatal closure targeting in the *pCAP1*-*CAP1*-*GFP* complementation lines (com-CAP1-GFP, S4A-S4D Fig), and discovered that the expression of *CAP1* in the *cap1–1* mutant rescued the phenotype of stomatal aperture in response to exogenous ABA (Fig 2C and S5A Fig). These results indicate that the *cap1–1* mutant is sensitive to the exogenous ABA. To further investigate the role of CAP1 in ABA signaling, we generated transgenic Arabidopsis overexpressing *CAP1* lines tagged with Flag (S4B Fig and S4E Fig). The analysis of drought tolerance showed that OE-*CAP1* (#23, #18 and #39) plants exhibited wilting status after exposure to drought stress for 10 days, and could not recover after rewatering (Fig 2D). OE-*CAP1* lines had significant lower levels of survival rate than Col-0 (Fig 2E). Accordingly, the water loss rate of detached rosette leaves in OE-*CAP1* lines were higher than that in Col-0 (Fig 2F). The analysis of stomatal aperture showed that all genotypes displayed fully open stomata in stomatal opening buffer, and OE-*CAP1* lines were compromised in response to ABA treatment with larger stomatal pores compared to Col-0 plants (Fig 2G and S5B Fig). These results suggest that CAP1 acts a negative role in response to drought stress and ABA-mediated stomatal closure.

### CAP1 is involved in ABA-induced stomatal closure through regulating actin reorganization

**Fig 3:**
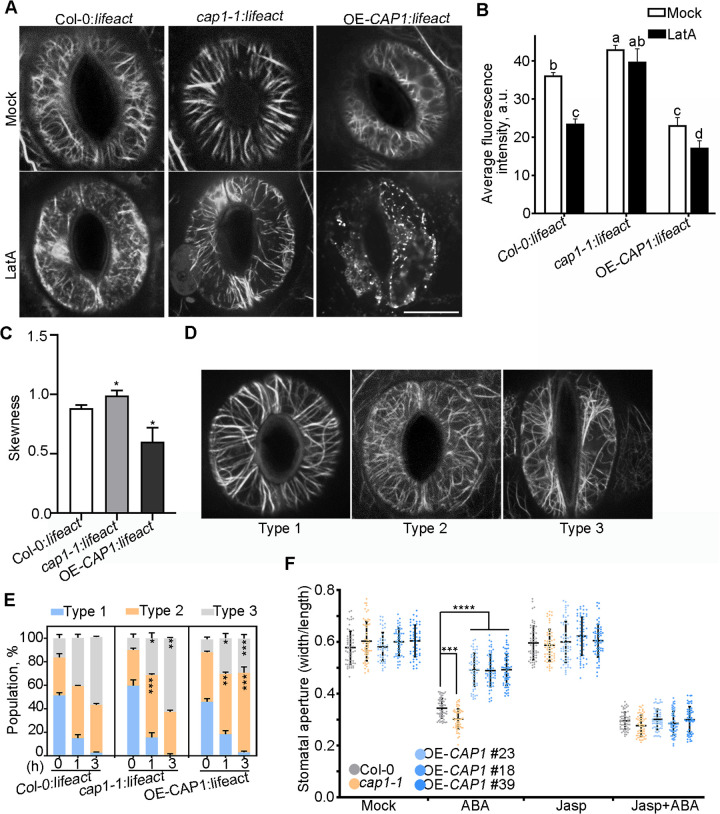
CAP1 is involved in stomatal closure via regulating actin organization. (A(A) Representative confocal images of actin networks in guard cells of transgenic plants harboring *ProUBQ::lifeact-GFP* before (Mock) and after 200 nM Latrunculin A (LatA) treatment. Scale bar = 10 µm. Col-0: *lifeact*, *cap1-1*:*lifeact* and OE-*CAP1*:*lifeact* indicate that Col-0, *cap1-1* and OE-*CAP1* #23 labelling filamentous actin. LatA, an inhibitor of actin polymerization. (B) Statistical analysis of filament average fluorescence intensity of leaves in (A) under normal condition (Mock) or LatA treatment. Values are mean ± SEM (Standard Error of the Mean) from three independent experiments (*n* = 3). Different letters denote statistical differences (*P* < 0.05) among samples as assessed by two-way ANOVA multiple comparisons. (C) Statistical analysis of skewness of leaves under normal condition in (A). Filaments in more than 20 guard cells were analyzed per genotype. Values are mean ± SEM from three independent experiments (*n* = 3). *P < 0.05 determined by Student’s *t*-test. (D) Representative confocal images of F-actin in *lifeact-*labeling guard cells during ABA-induced stomatal closure. The actin arrays are classified into Type 1 (well-organized radial array), Type 2 (random meshwork) and Type 3 (longitudinal array). (E) Histograms of the reorganization of F-actin in guard cells during stomatal closure induced by ABA. The population of three arrays was analyzed at indicated time after 20 µM ABA treatment. More than 50 stomata were analyzed per genotype. Values are mean ± SD (*n* = 3 biological repeats). Statistically significant differences (*P < 0.05; **P < 0.01; ***P < 0.001) were determined using Student’s *t*-test. (F) Stomatal closure induced by the Jasp and ABA treatment. Epidermal peel was pretreatment with 1 µM Jasp (or DMSO in control, Mock) for 30 min before adding 20 µM ABA at zero time on the graph and stomata were observed after 1 h treatment. Over 50 stomata were analyzed per genotype. Values are mean ± SD (*n* > 50). Statistically significant differences (***P < 0.001; ****P < 0.0001) were determined compared to Col-0 under the same conditions using one-way ANOVA with Tukey’s test. Jasp, Jasplakinolide, an actin-stabilizing agent.

To assess the effect of CAP1 on F-actin stability in guard cells, we generated transgenic plants harboring *ProUBQ::lifeact-GFP* (the actin marker) [33] in the background of *cap1–1* and OE-*CAP1* by crossing with Col-0:*lifeact,* respectively (S6A Fig). The drought assay showed that the F-actin marker did not alter plant tolerance to drought (S6B Fig). Quantification of skewness and density [17] showed that F-actin in *cap1–1:lifeact* guard cells under normal conditions were more stable, thick bundles (Fig 3A-3C and S1 Fig and S2 Video). This increased stability is consistent with the reduced actin dynamics reported for *cap1–1* pollen tubes [34]. In contrast, F-actin in OE-*CAP1:lifeact* guard cells displayed a thin and less bundled actin array (Fig 3A-3C and S3 Video)*.* These results indicate that CAP1 directly regulates actin organization in guard cells. After LatA treatment (an inhibitor of actin polymerization), F-actin in *cap1–1* guard cells mainly exhibited stable bundle structure or long fragment, while F-actin in OE-*CAP1* guard cells was seriously disassembled and the level of F-actin was significantly decreased (Fig 3A and 3B). These results indicate that CAP1 regulates F-actin stability during stomatal movement.

To investigate the role of F-actin stability in ABA-induced stomatal closure, we assessed actin reorganization under ABA treatment. According to the ratio of different types of actin arrays as previous characterized [17,35], the actin arrays displayed a well-organized radiated form (type 1) in opening stomata and then were disassembled into a random meshwork (type 2) in intermediate phase of stomatal closure, finally reassembled into longitudinally bundled form (type 3) in closed stomata (Fig 3D). Following ABA treatment, significantly higher levels of type 2 actin array (at 1 h) and the type 3 (at 3 h) were observed in *cap1–1* mutants, while OE-*CAP1* plants displayed lower levels of the type 3 compared to Col-0 plants (Fig 3E). These results demonstrate that the loss-of-function mutation of *CAP1* weakens actin turnover, thereby stabilizing actin arrays in the type 3 state to maintain stomatal closure and minimize water loss under drought stress. In contrast, overexpressing *CAP1* prevents the shift from the type 2 to the type 3, resulting in inhibiting of stomatal closure. Next, to determine if F-actin instability causes the retention of actin arrays in type 3 in OE-*CAP1* plants, we used Jasp to stabilize F-actin during stomatal closure. The data showed that the insensitivity of OE-*CAP1* plants to ABA during stomatal closure recovered to the level of Col-0 after Jasp pre-treatment (Fig 3F). This suggests that F-actin destabilization is partly responsible for the impaired ABA-induced stomatal closure. Collectively, our data reveal that CAP1 functions to disassemble F-actin in guard cells, thereby negatively regulating ABA-induced stomatal closure.

### The binding of RCAR12 to ABA releases the inhibition of CAP1 activity on F-actin disassembling

**Fig 4:**
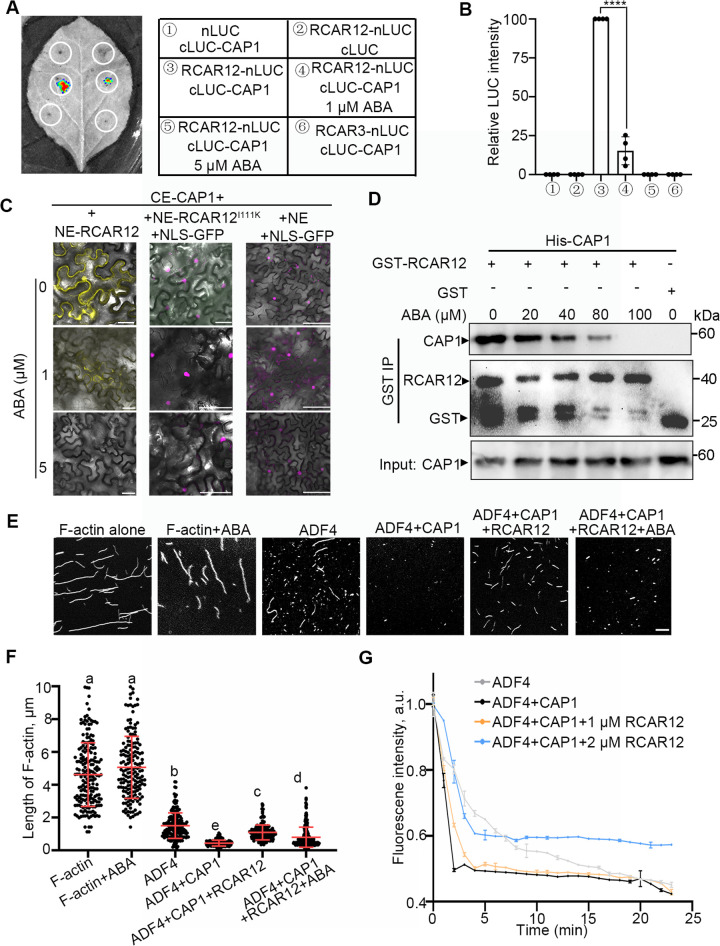
The inhibition of CAP1 activity by RCAR12 is released upon ABA binding to RCAR12. (A) Luciferase complementation imaging (LCI) assay. The indicated constructs were transiently expressed in *N. benthamiana* leaves infiltrated with 0, 1 or 5 μM ABA, respectively. Leaves were photographed 3-day after infiltration. (B) Statistical analysis of the relative fluorescence intensity values shown in (A). Values indicate mean ± SD (*n* = 4 biological repeats). Statistically significant difference (***P < 0.001) was determined using Student’s *t*-test. (C) The inhibition of the interaction of RCAR12 with CAP1 by the exogenous ABA in the BiFC assay. The exogenous ABA was added in infiltration buffer. NLS-mCherry was used as a control. RCAR12^I111K^ indicates a monomer. The signals of fluorescence were detected in the leaf epidermal cells of *N. benthamiana* after 3 days cultivation by a fluorescence microscope. At least three leaves and 3 plants were observed. Scale bars = 50 μm. (D) GST pull-down assay. The interactions supplemented with or without ABA were detected by immunoprecipitation with anti-GST and anti-His antibodies, respectively. Two biological experiments were performed with similar results (*n* = 2). (E) Representative confocal images of F-actin stained with Alexa488-Phalloidin. F-actin was incubated with indicated proteins for 30 min before staining. Scale bar = 5 μm. (F) Quantification of the length of F-actin in (E) by ImageJ. More than 150 filaments were measured for each treatment. Different letters indicate significant differences at *P* < 0.05, as determined by one-way ANOVA with Tukey’s multiple comparisons test. CAP1 (3 μM), ADF4 (3 μM), RCAR12 (1 μM), ABA (1 μM), F-actin (3 μM). (G) Depolymerization of F-actin (10% pyrene labeled). F-actin depolymerization was monitored by tracking the decrease in pyrene fluorescence per minute. a.u., arbitrary units. Values indicate mean ± SD (*n* = 3 biological repeats). CAP1 (3 μM), ADF4 (3 μM), RCAR12 (1 μM), ABA (1 μM), F-actin (3 μM).

ABA receptors belong to a class of START domain-like scaffolding proteins that undergo conformational changes upon binding ABA and then suppress the activity of PP2C phosphatases [36,37]. To examine the influence of ABA on the interaction between CAP1 and RCAR12, we performed a luciferase complementation imaging assay (LCI). After co-expression of RCAR12-nLUC and cLUC-CAP1 in *Nicotiana benthamiana* leaf cells, strong luminescence signals were observed in the absence of exogenous ABA, while the signals were significantly weakened when supplemented with 1 μM ABA and abolished with 5 μM ABA (Fig 4A and 4B). There was no luminescence signal after co-expression of RCAR3-nLUC and cLUC-CAP1 (Fig 4A), consistent with the results of BiFC (S1B Fig). The analysis by qPCR and immunoblot revealed that genes and proteins were expressed in leaves (S7A and S7B Fig). The BiFC assay showed that ABA treatment inhibited the CAP1-RCAR12 interaction, while having no effect on the fluorescence of the mCherry control (Fig 4C). These findings indicate that ABA is responsible for the reduction in fluorescence intensity. However, the RCAR12^I111K^ mutant, which is monomeric [38], consequently showed no interaction with CAP1 in tobacco epidermal cells (Fig 4C). Previous research has shown that exogenous ABA promotes the binding of dimeric ABA receptors to ABI1 (clade A protein phosphatase 2C) by triggering a conformational rearrangement in dimeric RCAR12, which induces receptor dissociation [37,39]. Here, GST-Pull down assay showed that prokaryotic expressed GST-RCAR12 pulled down His-CAP1, while His-CAP1 band was attenuated to some extent in the presence of ABA (Fig 4D). Taken together, these results suggest that ABA adding inhibits the interaction of CAP1 and RCAR12.

Next, we examined the ability of CAP1 on binding F-actin after interacting with RCAR12. A high-speed co-sedimentation assay revealed that CAP1 was coprecipitated with F-actin in the pellets, while the negative control BSA protein was present primarily in the supernatant (S8A Fig). However, the F-actin-binding ability of CAP1 was weakened in the presence of RCAR12, but not affected by exogenous ABA (S8B Fig and S8C Fig). In combination of florescent signals in the complementation line (S4C Fig), CAP1 colocalized with F-actin (S4D Fig), indicating that it is an actin-binding protein. Because CAP1 had no significant effect on the distribution of actin in supernatant and precipitation, it has a limited effect on actin disassembly in vitro (S8B Fig and S8C Fig).

In Arabidopsis, The activity of CAP1 in actin severing and depolymerization requires actin depolymerization factor ADF [31,34,40,41], thus pointing to a synergistic role of CAP1 and ADF in F-actin disassembling. Given that ADF4 is involved in drought tolerance in Arabidopsis [15], we hypothesized a functional link with CAP1. To this end, we tested their interaction in plant cells. This interaction was confirmed by the BiFC assay (S9A Fig). Next, the visualization of F-actin labeled with Alexa-488-phalloidin showed that ADF4 alone disassembled F-actin into short filaments and the addition of CAP1 resulted in shorter filaments (Fig 4E and 4F). This finding supports the established role of CAP1 in enhancing ADF-mediated F-actin depolymerization [42]. However, the average F-actin length following co-incubation of RCAR12, CAP1 and ADF4 was longer than with the CAP1-ADF4 complex but shorter than with the exogenous ABA supplementation (Fig 4E and 4F). To monitor the dynamic process, pyrene F-actin disassembly assays were performed. The data indicated that F-actin disassembly mediated by the CAP1-ADF4 complex was significantly inhibited by RCAR12 in a dose-dependent manner (1–2 μΜ RCAR12), while RCAR12 or CAP1 alone did not disassemble F-actin (Figs 4G and S9B Fig). Collectively, our in vitro results suggest that while RCAR12 can inhibit the F-actin disassembling activity of the CAP1-ADF4 complex, ABA adding acts to alleviate this inhibition.

Previous studies have shown that a mutation at M319 site of CAP1 weakens its interaction with RCAR8 [24]. Given this context, we examined whether the M319 mutation likewise disrupts the interaction with RCAR12 using an LCI assay. The results showed that the luminescence signal was stronger in the leaves co-expressing RCAR12-nLUC and cLUC-CAP1 than co-expressing RCAR12-nLUC and cLUC-CAP1^M319A^ (S10A and S10B Fig). The measurement of the length of F-actin in vitro demonstrated that co-incubation of CAP1^M319A^ and ADF4 disassembled F-actin into short fragment, which was not affected by the addition of RCAR12 (S10C Fig and S10D Fig). Thus, our results suggest that the repression of RCAR12 on CAP1-ADF4 activity in vitro actin filaments is partially dependent on the M319 site of CAP1.

### OST1-mediated phosphorylation of CAP1 inhibits its binding to ADF4

**Fig 5:**
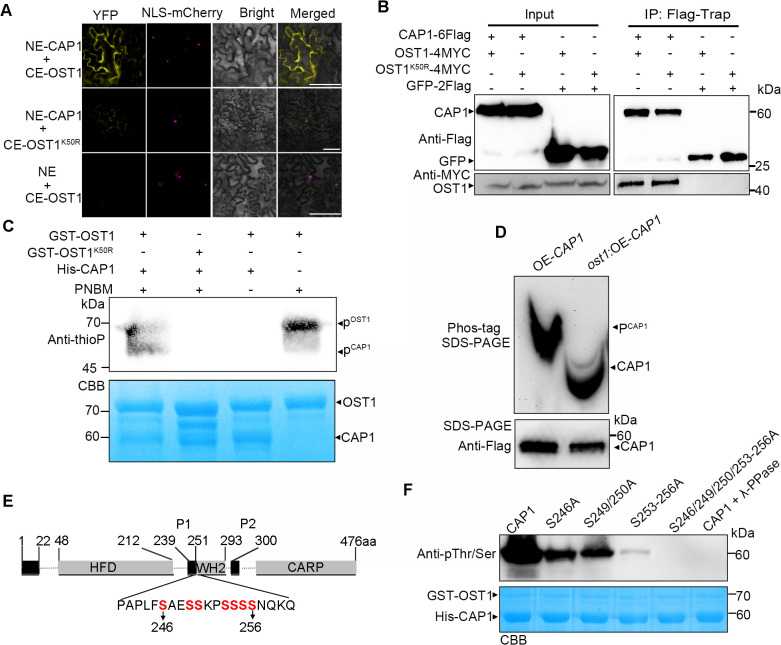
OST1 physically interacts with CAP1 and phosphorylates at Ser246/249/250/253/254/255/256 residues of CAP1. (A) Interaction between OST1 and CAP1 validated by BiFC assays. The images are the representative of three independent experiments (*n* = 3). OST1^K50R^ indicates the kinase-dead form of OST1. NLS-mCherry indicates nuclear localization. We repeated the experiment four times with similar results and at least 8 leaves were observed per time. Scale bars = 50 μm. (B) Co-IP assay was performed to test the interaction of CAP1 with OST1 and OST1^K50R^, detected with anti-Flag and anti-MYC antibodies. GFP-2Flag was set as a negative control. The images are the representative of two independent experiments (*n* = 2) with the same results. (C) In vitro kinase assay showing the phosphorylation of CAP1 by OST1. The phosphorylation of His-CAP1 was examined using an anti-thiophosphate ester (thioP). The loading amounts of His-CAP1 and GST-OST1 were detected using Coomassie Brilliant Blue (CBB) staining. (D) OST1 phosphorylates CAP1 *in vitro*. CAP1 from OE-*CAP1* and *ost1*:OE-*CAP1* seedlings was immunoprecipitated and separated by a Pho-tag-PAGE gel, then analyzed with anti-Flag antibody. The IP input was analyzed by regular SDS-PAGE gel with anti-Flag antibody. (E) The phospho-sites of CAP1 identified from mass spectrometry analysis. (F) Phosphorylation levels of mutation of phosphosites (serine to alanine, S to A) by OST1, detected with anti-phosphoserine antibody. The loading amounts of His-CAP1 and GST-OST1 were detected by CBB staining. Values indicate mean ± SD (*n* = 3 biological repeats). CAP1 (3 μM), ADF4 (3 μM), RCAR12 (1 μM), ABA (1 μM), F-actin (3 μM).

Under drought stress, the accumulation of ABA rescues CAP1’s activity inhibited by RCAR12 (Fig 4). To maintain stomatal closure, however, CAP1’s activity on reassembling F-actin may be inhibited. This could be achieved through phosphorylation as reported that the phosphorylation of CAP affects its association with ADF and consequently remodel the actin cytoskeleton in animals [42]. Because OST1 kinase is activated in guard cells under drought stress [43], we specifically assessed whether OST1 phosphorylates CAP1 in Arabidopsis. BiFC and CoIP assays confirmed the interaction between OST1 and CAP1 in vivo (Fig 5A and 5B). In addition, as a Lys-50 residue of OST1 is essential for ATP binding [44,45], we generated kinase-dead OST1^K50R^ [46] ‌attenuated the fluorescent signals (Fig 5A). Nevertheless, the Co-IP assay showed that OST1^K50R^ interacted with CAP1, similar to wild-type OST1. This discrepancy is possible because the proteins were expressed in separate cell systems. To assess the phosphorylation of CAP1, an in vitro kinase assay was performed. The results showed OST1 phosphorylated CAP1, but the kinase-dead form OST^K50R^ did not (Fig 5C), confirming that the mutant version OST1^K50R^ lacks kinase activity. To validate the in vivo phosphorylation of CAP1 by OST1, we first generate the *ost1*:OE-*CAP1*:*lifeact* plants by crossing (S11 Fig). Then, to directly assess CAP1 phosphorylation, we performed an immunoblotting assay using Phos-tag SDS-PAGE. The results showed that CAP1 protein displayed slower mobility in *ost1*:OE-*CAP1*:*lifeact* compared to that in OE-*CAP1*:*lifeact* (Fig 5D). These results demonstrate that OST1 phosphorylates OST1.

Since the kinase activity of OST1 was activated by ABA [47], we therefore investigated OST1 phosphorylates CAP1. LC-MS/MS analysis identified that a cluster of the serine (Ser) 246, 249, 250, 253, 254, 255 and 256 as potential CAP1 phosphorylation sites mediated by OST1. To determine the phosphorylation sites by OST1 on CAP1, we purified various CAP1 variants, including His-CAP1^S246A^, His-CAP1^S249/250A^, His-CAP1^S253/254/255/256A^ (His-CAP1^S253-256A^), His-CAP1^S246/249/250/253-256A^. In vitro phosphorylation assays showed that OST1 could phosphorylate CAP1, CAP1^S246A^, CAP1^S249/250A^, and CAP1^S253-256A^ (Fig 5E and 5F). However, the phosphorylation signal of CAP1^S253-256A^ was significantly reduced and CAP1^S246/249/250/253-256A^ was abolished (Fig 5E and 5F). To verify the in vivo phosphorylation of these residues, phosphorylation profiling was performed in OE-*CAP1* and *ost1*:OE-*CAP1* seedling after ABA treatment. The phosphopeptide signal at Ser246/249/250/253–256 in *ost1*:OE-*CAP1* was significantly lower than that in OE-*CAP1* (S12A Fig). These results suggest that serine residues of Ser246/249/253/254/255/256 are critical phosphosites of CAP1 mediated by OST1.

**Fig 6:**
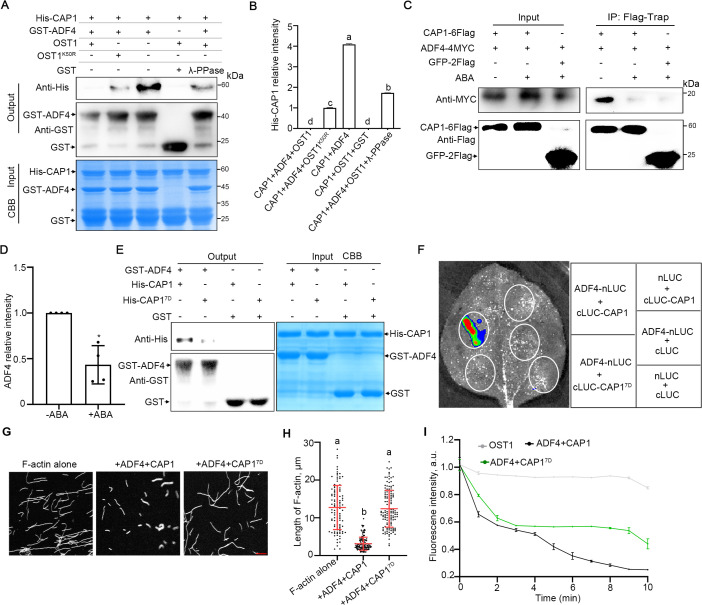
The phosphorylation of CAP1 by OST1 suppresses the interaction of CAP1 and ADF4. (A(A) GST pull-down assay was performed for the interaction of ADF4 with CAP1. In vitro phosphorylation assay was performed, followed by GST pull-down. OST1^K50R^ indicates the kinase-dead form of OST1. λ-PPase, Lambda Protein Phosphatase. The loading amounts of His-CAP1, GST-ADF4 and GST were detected by CBB staining.(B) Relative protein levels of His-CAP1 in (A). The intensity of His-band was measured using ImageJ software. Three independent experiments were provided for the data statistics. Values are means ± SD (*n* = 3). Different letters indicate statistically significant differences by one-way ANOVA with Tukey’s test (*P* < 0.05). (C) Analysis of the interaction of CAP1 using Co-IP assay before and after 50 μΜ ABA treatment for 3 h. CAP1-6Flag was immunoprecipitated using anti-Flag-M2 agrose, and immunoblotting assays were performed using anti-Flag and anti-MYC antibodies. (D) Relative protein levels of ADF4 in (C). The intensity was measured using ImageJ software. Four independent experiments were provided for the data statistics. Values are means ± SD (n = 4). Asterisk indicates statistically significant differences (*P < 0.05, Student’s *t-*test, two-sided). (E) The interactions of the phosphomimic variant of CAP1^S246/249/250/253-256D^ and CAP1 with ADF4 were analyzed using in vitro GST pull-down assay. The loading was detected by CBB staining. (F) The interactions of the phosphomimic variant of CAP1^S246/249/250/253-256D^ and CAP1 with ADF4 were analyzed using LCI (Luciferase Complementation Imaging) assay. The indicated constructs were transiently expressed in *N. benthamiana* leaves. Leaves were photographed 3-day after infiltration. (G) Representative confocal images of F-actin. F-actin was incubated with indicated proteins for 30 min before staining with Alexa488-Phalloidin. Scale bar = 5 μm. (H) Quantification of the length of F-actin in (G) by ImageJ. More than 100 filaments were measured for each treatment. Values are mean ± SEM. *P < 0.05, as determined by one-way ANOVA with Tukey’s test. (I) Depolymerization of F-actin (10% pyrene labeled). F-actin disassembly was monitored by tracking the decrease in pyrene fluorescence per minute. a.u., arbitrary units. Values indicate mean ± SD (*n* = 3 biological repeats). CAP1 (3 μM), ADF4 (3 μM), OST1 (1 μM), F-actin (3 μM).

Based on the finding that CAP1 phosphorylation loses its association with cofilin in human signaling [42,48], we hypothesized a conserved regulatory mechanism in plants. We thus investigated OST1’s influence on the CAP1-ADF4 complex via a GST pull-down assay. His-CAP1 was first phosphorylated by OST1 and then incubated with GST-ADF4 or GST. Our results showed that phosphorylation abolished CAP1 binding to ADF4; this binding was reduced when CAP1 was incubated with kinase-dead OST1, and could be partially rescued by treatment with Lambda Protein Phosphatase (λ-PPase) (Fig 6A and 6B). To further verify in vivo relation of OST1-CAP1-ADF4, we performed the BiFC assay. YFP fluorescent signals and protein levels were significantly attenuated in tobacco leaves co-expressing OST1, CE-ADF4 and NE-CAP1, compared to co-expressing NE-CAP1 and CE-ADF4 (S12B Fig). In addition, we found that ABA attenuated the interaction of ADF4 with CAP1 (Fig 6C and 6D). To identify the role of CAP1 phosphorylation, we generated phosphomimetic mutant CAP1^S246/249/250/253-256D^ (CAP1^7D^) using site-directed mutagenesis. The GST pull-down and LCI assays showed that the interaction between ADF4 and CAP1^7D^ was abolished (Fig 6E and 6F). These results indicate that OST1-mediated phosphorylation of CAP1 represses its binding to ADF4.

### OST1-mediated phosphorylation of CAP1 inhibits its F-actin disassembling activity

To determine the function of OST1-mediated CAP1 phosphorylation on F-actin, Alexa488-Phalloidin was used to label F-actin. The pre-polymerized F-actin was disassembled into dots after co-incubation with CAP1 and ADF4, while phospho-mimetic CAP1^7D^ inhibited the actin disassembly (Fig 6G and 6H). The pyrene F-actin disassembly assay further revealed that the activity of CAP1^7D^-ADF4 complex on disassembling F-actin was weakened compared to the CAP1 + ADF4 complex, while OST1 alone did not reduce the fluorescence intensity of pyrene (Fig 6I). These results suggest that CAP1 phosphorylation mediated by OST1 represses the CAP1-ADF4 activity on actin depolymerization.

**Fig 7:**
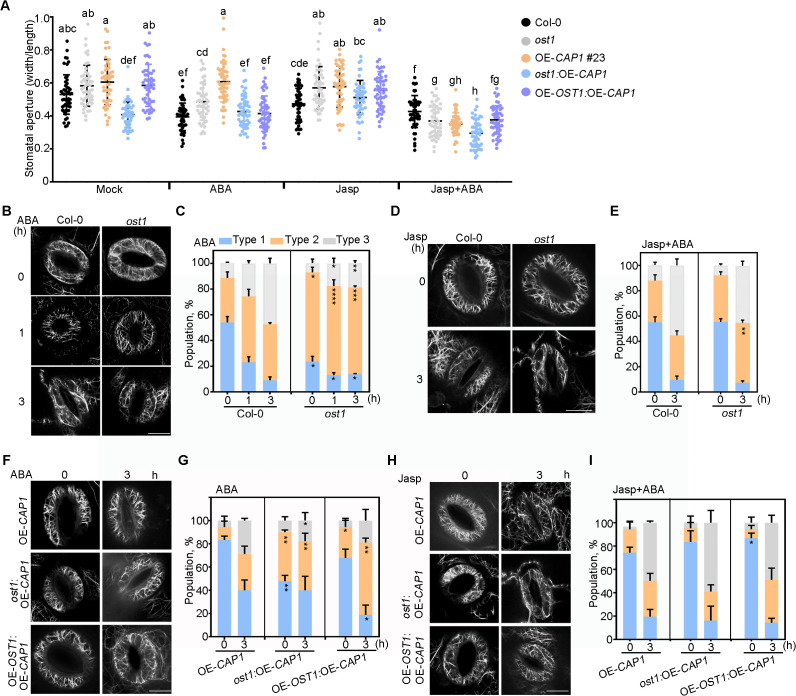
The phosphorylation of CAP1 by OST1 regulates actin-disassembling in guard cells. (A) The measurements of stomatal aperture. Leaves were incubated in opening buffer without (Mock) and with 20 μM ABA treatment for 3 h, or pretreatment with 1 µM Jasp for 30 min before adding 20 µM ABA. Over 50 stomata were analyzed per genotype. Letters above bars indicate significant differences according to one-way ANOVA Tukey’s multiple comparisons test (*P* < 0.05). The experiment was repeated three times with similar results. (B) F-actin status in Col-0 and *ost1* mutant before and after 20 µM ABA treatment for 1 h and 3 h. (D) F-actin status in Col-0 and *ost1* mutant by 20 µM ABA treatment with 1 µM Jasp pretreatment for 30 min. (F) F-actin status in OE-*CAP1*, *ost1*:OE-*CAP1* and OE-*OST1*:OE-*CAP1* plants before and after 20 µM ABA treatment for 3 h. (H) F-actin status in OE-*CAP1*, *ost1*:OE-*CAP1* and OE-*OST1*:OE-*CAP1* plants by 20 µM ABA treatment with 1 µM Jasp pretreatment for 30 min. (C, E, G, I) Histograms of the reorganization of F-actin in guard cells during stomatal closure according to the classification in Fig 3D in (B, D, F, H), respectively. The experiments were repeated three times and the values represent the average mean ± SD of three independent experiments with at least 30 guard cells measured at each indicted time point per each phenotype. Statistically significant differences (*P < 0.05; **P < 0.01; ***P < 0.001) were determined using Student’s *t*-test. Bars, 5 µm. Asterisks indicate significant differences in percentage of type 3 guard cells by the Student *t*-test (*P < 0.05; **P < 0.01, compared to the wild-type Col-0 in (C, E), and OE-*CAP1* in (G, I), respectively..

To analyze the genetic relationship between OST1 and CAP1, we generated OE-*OST1*:OE-*CAP1* expressed lifeact-GFP (S11 Fig). The data showed that stomatal aperture of OE-*OST1*:OE-*CAP1* had no significant difference compared to Col-0, but smaller than that of OE-*CAP1* leaves after exogenous ABA treatment (Fig 7A). These results indicate that overexpression of *OST1* eliminates the inhibition of stomatal closure of OE-*CAP1* plants. However, it is interesting that the stomatal aperture of *ost1*:OE-*CAP1* displayed no significant difference compared to Col-0 before and after ABA treatment (Fig 7A), suggesting that the *ost1*:OE-*CAP1* plants were insensitive to the exogenous ABA. In addition, Jasp significantly increased stomatal aperture in *ost1*:OE-*CAP1* plants, and the stomatal aperture of OE-*OST1*:OE-*CAP1* resumed to Col-0 upon a mix of Jasp and ABA treatment (Fig 7A), indicating that overexpressing *OST1* could rescue ABA-induced stomatal closure in OE-*CAP1*.

Next, we analyzed the resistance of F-actin to LatA treatment, and found that F-actin in guard cells was more sensitive to LatA treatment in *ost1:lifeact* than Col-0*:lifeact* (S13A and S13B Fig). After ABA treatment, the population level of type 3 in *ost1* was significant lower, but type 2 was higher than that in Col-0 plants (Fig 7B and 7C). When treated with Jasp followed by ABA, however, *ost1* mutants had similar type 3 actin array with Col-0 plants (Fig 7D and 7E), suggesting that Jasp could rescue the ABA-insensitive stomatal closure of *ost1* mutants. In the presence of ABA, OE-*OST1*:OE-*CAP1* plants exhibited faster actin reorganization than OE-*CAP1*. Without ABA treatment, *ost1*:OE-*CAP1* plants displayed a higher level of type 2 actin arrays but a lower of type 1 compared to OE-*CAP1* plants (Fig 7F and 7G), accounting for their smaller stomatal aperture under normal conditions (Fig 7A). When treated with ABA, *ost1*:OE-*CAP1* plants displayed a higher levels of type 2 and lower of type 3 actin arrays compared to OE-*CAP1* plants (Fig 7F and 7G). However, actin reorganization was rescued in guard cells of OE-*OST1*:OE-*CAP1* exposed to Jasp, followed by ABA treatment (Fig 7H and 7I). The data suggest that OST1 inhibits the activity of CAP1 and works in the final phase of stomatal closure, through mediating the reassembly of F-actin.

## Discussion

As the important cyto-architectures in guard cells, F-actin is involved in stomatal movement by structural changes [49]. Emerging studies reveal that the dynamic of F-actin and ABA synergize to regulate the process of stomatal closure and opening. For example, the phosphorylation of ADF4 by CKL2 inhibits its activity on F-actin disassembly and then stabilizes F-actin in stomatal closure under drought stress, whereas CKL2 is repressed by the PP2C phosphatase ABI1 (ABA Insensitive 1) and ABI2 in ABA-dependent manner, forming the feedback regulation [15,22]. Although the reassembly of F-actin during stomatal closure is documented, the underlying mechanisms responsible for F-actin assembly and disassembly by ABA remain to be characterized. In this study, we provide new mechanistic insight into ABA-induced stomatal closure through CAP1’s control of actin turnover.

Arabidopsis CAP1 is essential for plant growth and development [50,51]. Mutation or knockdown of CAPs in yeast, mouse and plants leads to out-of-balance of actin cytoskeleton dynamics, including aberrant cell morphology and polarity [34,52–54]. Small leaves in the *cap1–1* mutants are one factor responsible for their strong drought tolerance (Fig 1A). However, there is no phenotypic difference between Col-0 and overexpressing *CAP1* plants under normal conditions, thereby the actin organization contributes to the insensitivity of ABA-induced stomatal closure in OE-*CAP1* plants under drought stress. Taken together, CAP1 mediates leaf development and actin turnover in guard cells in response to drought stress.

The interaction between CAP1 and RCAR12 required WH2 or CARP domain, both of which are essential for CAP1’s oligomerization (Fig 1D). It is consistent with the analysis of crystal structure [40], exhibiting an oligomerization in favor of interacting with the dimeric ABA receptors, including RCAR11, RCAR12 and RCAR14. Consequently, binding of dimeric RCARs to the WH2 domain of CAP1 may inhibit the delivery of ADF4-actin from the N- to C-terminus of CAP1. This likely results in the retention of ADF4 in the complex, thereby inhibiting dynamic reorganization of the actin cytoskeleton [40]. Additionally, RCAR12 binding to the CARP domain of CAP1, may also interfere with ADP-G-actin binding and subsequently represses nucleotide exchange on actin monomers [54–56].

Evidence suggests that CAP1 and ABA receptors function synergistically to regulate seedling development. CAP1 normally associates with monomeric RCAR8 (PYL5), which is essential for CO2-induced stomatal closure [6,24]. While single ABA receptor mutants show no distinct growth phenotype [5,57], loss-of-function of CAP1 (*cap1–1*) or multiple receptors (PYR1/PYL1/2/4/5/8, corresponding to RCAR11/12/14/8/3) are dwarfed [51,58]. Because the loss of multiple, but not single, receptors leads to a growth defect in Arabidopsis, functional redundancy among the receptors is implied. Thus, CAP1 appears to cooperate with this redundant receptor network to control development.

Under dehydration conditions, ABA quickly accumulates in guard cells, and the dimeric RCAR12 is phosphorylated and binds to ABA [59]. ABA receptors binding to ABA associate with PP2Cs, in turn, PP2Cs release OST1 kinase activity, thereby phosphorylating the slow anion channel (SLAC1) [60,61]. The activation of SLAC1 triggers anion efflux from guard cells, resulting in membrane depolarization, and subsequently induces stomatal closure [62]. In this study, we found that CAP1 releases from RCAR12 binding to ABA, resumes its activity on actin disassembly in guard cells (Fig 4). The dimeric state of RCARs, which is inactive or less active, is phosphorylated by CARKs induced by ABA [59]. Subsequently, these dimers undergo structural changes and break apart into monomers, resulting in a higher ABA binding affinity. OST1 is quickly activated and then phosphorylates CAP1 (Fig 4C and 4D). Then, CAP1 initiates through F-actin-mediated stomatal closure, shifts actin array from type 1 to type 2 in response to ABA, which is consistent with the actin-related proteins ARP2/3 complex regulating the remodeling of stomatal movement [12].

Additionally, the *cap1–1* and *adf4* mutants display small stomatal aperture and high skewness value (Fig 3A-3C), indicating that loss-of-function of *CAP1* or *ADF4* increases the extent of actin filament bundling in guard cells [15]. In CAP1-deficient hippocampal neurons, super-resolution and live-cell imaging reveal that impaired synaptic F-actin organization and dynamics correlate with altered spine morphology [63]. Similarly, *CAP1* overexpression reduced actin mesh levels, and CAP1 interacts with ADF/cofilin to facilitate actin depolymerization during mouse oocyte maturation [64]. Here, our findings demonstrate that the CAP1-ADF4 complex triggers actin depolymerization (Fig 4F), indicating that CAP1 and ADF4 have similar function on cytoskeleton-mediated stomatal closure during drought stress. In the in vitro F-actin assay, data indicate that CAP1 depends on ADF4 to regulate F-actin depolymerization (Fig 4E-4G). Previous study shows that ADF4 activity is regulated by CKL2 during ABA-mediated stomatal closure [15]. Additionally, ABA-dependent ABI1 and ABI2 inhibit CKL2 kinase activity, thereby forming a feedback loop that regulates ABI1 stabilization [22]. There, we infer that CAP1 acts synergistically with ADF4 in actin remodeling to regulate ABA-mediated stomatal closure.

Previous study reveals that phosphorylation of mouse CAP1 inhibits its binding to the cofilin and phosphoregulation kinase is crucial for CAP1 functions in regulating the actin cytoskeleton rearrangement and cancer cell invasiveness [48]. However, the phosphorylation sites of CAP1 mediated by OST1 are located in the WH2 domain (Fig 5F). This is different from the phosphorylation of mouse CAP1 at Ser308/310 by GSK3, locating in CARP domain [48]. Different phosphosites on a protein typically determine the specificity of downstream signaling [65]. Consequently, the distinct phosphosites of CAP1 in plants versus animals specify its function in stomatal cells in planta.

Here, we found that the *ost1*:OE-*CAP1* plants exhibit smaller stomatal aperture than *ost1,* OE-*CAP1* and Col-0 plants under normal condition (Fig 7A). However, there is no significant difference among *ost1*, *cap1–1,* OE-CAP1, and Col-0 (Figs 1C, 1D and 7A). It is possible that a high level of dephosophorylation of CAP1 inhibits stomatal opening. In this study, OST1-mediated phosphorylation of CAP1 inhibits the activity of the CAP1-ADF4 complex on actin cytoskeleton (Fig 6G-6I). It is likely due to inhibit the association between ADF4 and phosphorylated CAP1 (Fig 6E and 6F). Therefore, CAP1-mediated reorganization of stomatal actin cytoskeleton is controlled by phosphorylation.

**Fig 8:**
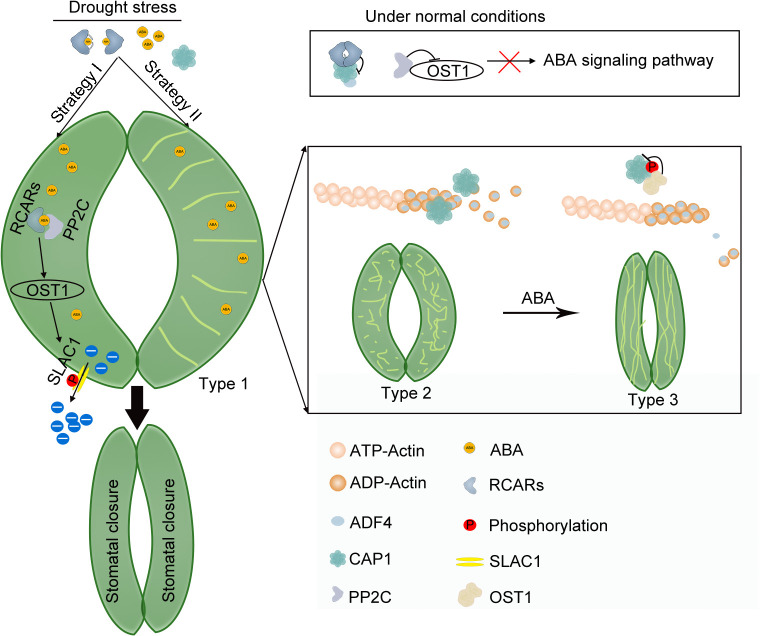
A proposed working model of F-actin disassembly during ABA-mediated stomatal closure in Arabidopsis. CAP1 interacts with ABA receptor RCAR12, and the interaction inhibits its activity on F-actin disassembly whilst OST1 inhibited by the protein phosphatase type 2C (PP2C) suppresses ABA signal transduction pathway under normal conditions (Type 1). Under drought stress, CAP1 releases from RCAR12 after binding to ABA, and promotes F-actin disassembly (Type 2). Upon ABA binding, dissociation of the RCAR12 dimer, inhibits PP2C activity, and releases OST1 activity. The activation of OST1 leads to the latter series of events. Firstly, OST1 phosphorylates the SLAC1 channels as previous reported [56]. Secondly, OST1 phosphorylates CAP1 to inhibit actin depolymerization. F-actin in guard cells display the longitudinal array to maintain stomatal closure in Arabidopsis (Type 3).

The dynamic balance between disassembly and reassembly of F-actin is essential for maintaining plant growth-in particular, stomatal movement. Under normal conditions, the dimeric RCARs interact with CAP1 and inhibit the disassembly activity of the CAP1-ADF4 complex in guard cells. Under drought stress, the dimeric RCARs are disassociated after ABA adding [37,66], leading to activate ABA signaling in response to drought stress. At the same time, CAP1 after released from the dimeric RCARs disassembles F-actin, resulting in the shift of the well-organized cortical filaments in the guard cells of open stomata to randomly distributed filaments. Then, ABA-activated OST1 inhibits the activity of CAP1-ADF4 complex, which finally causes filaments to reorganize into highly bundled long cables in the longitudinal direction for stabilization of closed stomata (Fig 8). In summary, ABA, the core components of ABA signaling and CAP1-ADF4 complex synergistically mediate stomatal closure in response to drought stress in *A. thaliana*.

### Limitations of the study

This study shows that the CAP1-ADF4-OST1 module regulates ABA-mediated stomatal closure. However, due to the developmental defects and pollen tube growth defects of *cap1–1* mutants [34], we failed to obtain the *cap1–1adf4*, *cap1–1ost1* double mutants, the complementation of a non-phosphorylatable (dephospho-mimetic) CAP1^S246/249/250/253/254/255/256A^ and a phosphor-mimetic variant CAP1^S246/249/250/253/254/255/256D^ to further analyze the stomatal closing processes. Additionally, due to the close target residues of CAP1 by OST1, we did not figure out which residue or combination plays a predominant role in regulation of stomatal movement. These limitations highlight the need for further research to the process of ABA-induced stomatal closure for elucidating the mechanism of drought responses in Arabidopsis.

## Materials and methods

### Plant materials and growth conditions

*Arabidopsis* plants Col-0, *cap1–1* (Salk_112802), *ost1* (Salk_008068) [67], OE-*OST1* using *35S* promoter and *UBQ10::lifeact-GFP* (*lifeact*) were used in this study. All primers used for the T-DNA mutant identification were listed in S1 Table. *N. benthamiana* and *Arabidopsis* plants were grown at 24°C under 60% relative humidity with a photoperiod of 16-h light/8-h dark (100 μmol m^-2^ s^-1^). For plate culture, seeds were surface sterilized and germinated on solid MS medium containing 2% sucrose and 0.8% agar, pH 5.8.

### Transgenic plant lines

*CAP1* CDS (coding sequence) was cloned into *p*CAMBIA2300 to obtain *Pro35S*::*CAP1-flag* construct. *RCAR12* CDS was cloned into pEarleyGate100 tagged with GFP. The pCAP1-gCAP1 sequence, in which the *CAP1* CDS is driven by the 1000-bp *CAP1* promoter, was synthesized (Tsingke, Beijing, China) and cloned into the *p*CAMBIA1301-NOS binary vector. Subsequently, the vector was introduced into cap1–1 T-DNA mutants to generate the CAP1 complementation lines. Transgenic plants were screened for kanamycin, hygromycin or Basta resistance. All primers for creating transgenic plants were listed in the S1 Table.

### Yeast two-hybrid assay

Plasmids *p*GBKT7 containing *RCAR1–14* [59] and *p*GADT7-rec containing *CAP1* were co-transformed into yeast strain AH109 cells following standard heat-shock protocols. Then serially diluted (10^-1^, 10^-2^ and 10^-3^) yeast cultures were placed on SD medium lacking leucine (Leu), tryptophan (Trp), histidine (His) and adenine (Ade) (SD/-Leu/-Trp/-His/-Ade) at 30°C for 3 d.

### Bimolecular fluorescence complementation (BiFC) assay

The CDS of *CAP1*, *OST1* and *OST1*^*K50R*^ were cloned into the binary *p*SPYNE (NE) or *p*SPYCE (CE) vector to generate NE-CAP1, CE-OST1 and CE-OST1^K50R^, respectively. CE-CAP1 and NE-RCAR3/8/11/12/13/14 vectors were previously descripted [24,59]. Then, the vectors were introduced into *Agrobacterium tumefaciens* strain GV3101. Before infiltration into *N. benthamiana* leaves, cells were resuspended in infiltration buffer (0.2 mM acetosyringone, 10 mM MgCl_2_, and 10 mM MES at pH 5.6) to identical concentrations (OD_600_ = 1.0). After 3 d, leaves were observed and photographed with a confocal laser-scanning microscope.

## Co-IP assay

For Co-IP assays, RCAR12 was cloned into the *p*CAMBIA1302 to generate *p*CAMBIA1302-RCAR12-GFP. OST1 and the mutation of OST1^K50R^ were cloned into the *p*CAMBIA1307 to generate *p*CAMBIA1307-OST1/OST1^K50R^-4MYC. The *p*CAMBIA1307-ADF4–4MYC was previously descripted [67]. The *p*EarleyGate100-GFP-2Flag vector expressing GFP-2Flag was used a negative control. The vectors were transiently expressed in tobacco cells by Agrobacterium-mediated infiltration. After 2-d infiltration, 2 *g* of agro-infiltrated *N. benthamiana* leaves was ground in 5 mL lysis buffer (25 mM Tris-HCl [pH 7.5], 150 mM NaCl, 5 mM EDTA, 0.5% Triton X-100, 2% PVPP, 2 mM DTT, 10% glycerol, 1x cOmplete Protease Inhibitor Cocktail). The samples were vigorously for 1 min and incubated in an ice bath for 30 min. The mixture was centrifuged twice at 15000 *g* for 30 min at 4°C. After filtered through cheesecloth, the supernatant was incubated with anti-GFP or anti-Flag (Transgen, Beijing, China) agarose beads with gentle rotation at 4°C for 4 h. After incubation, the beads were washed four times with washing buffer (50 mM Tris-HCl [pH 7.5], 100 mM NaCl, and 1mM EDTA). The samples were diluted with SDS-PAGE loading buffer and boiled at 95°C for 10 min. The proteins were separated by 10% SDS-PAGE.

### Split-luciferase assay

The luciferase complementation imaging (LCI) assay was performed as previously descripted [68]. The CDS of *RCAR12*, *RCAR3*, *CAP1* and *CAP1*^*M319A*^ were cloned into the *p*CAMBIA1300-cLUC or *p*CAMBIA1300-nLUC vector to generate RCAR12/3-nLUC, cLUC-CAP1/CAP1^M319A^ vectors, respectively. The CAP1^M319A^ mutation was previously descripted [24]. The constructs were transformed into GV3101, which were injected into *N. benthamiana* leaves. After 3 d, 1 mM luciferin was sprayed onto the leaves for CCD imaging (PerkinElmer IVIS Lumina III).

### Stomatal aperture assay

To analyze the stomatal aperture, the *ost1* and OE-*OST1 plants* crossed with OE-*CAP1* to generate *ost1*:OE-CAP1; OE-*OST1*:OE-*CAP1* genotypes. Leaf peels were collected from the abaxial side of 3-week-old plant rosette leaves, and immersed in opening buffer (50 mM KCl, 50 μM CaCl_2_, and 10 mM MES-KOH, pH 6.15) at 24°C for 3 h. Then, leaf peels were treated with or without ABA, Jasp for indicated time. Stomata were observed after treatment with 20 μM ABA for 0, 1 h (Figs 2C, 2G, 3F, and 7A). Actin filament patterns in guard cells were scored following treatment with 20 µM ABA for 0, 1 h and 3 h in Figs 3E and 7B; for 0 and 1 h in Fig 7D-7I. Stomatal apertures (width/ length) of more than 50 stomata from each line were measured using ImageJ.

### Water loss and drought tolerance analysis

For water loss analysis, five detached rosette leaves of each genotype from 3-week-old plants were weighed at the indicated times (accumulated more than 6 h). For drought tolerance analysis, 3-week-old seedlings grown in pots were subjected to drought stress treatment by water withholding for 15 days, followed by normal water supply for another 2 days. The morphological changes of plants were recorded.

### Biochemical characterization of CAP1

The purification of His-ADF4 was previously descripted [69]. All proteins were dialyzed against buffer G (5 mM Tris-HCl, 0.2 mM DTT, pH 7.0) and centrifuged at 100,000 *g* at 4°C for 30 min. Actin was prepolymerized in 1 X KMEI buffer (50 mM KCl, 1 mM MgCl_2_, 1 mM EGTA, 10 mM imidazole, pH 7.0) at room temperature for 1 h. High-speed cosedimentation assay and pyrene-actin depolymerization assay were performed as previously described [35].

### Visualization of actin in guard cells

To observe actin dynamics in guard cells, the probe Lifeact-GFP was introduced into *cap1–1*, OE-*CAP1*, *ost1*, OE-*OST1* plants via crossing with Col-0 Arabidopsis plants expressing *ProUBQ::Lifeact-GFP*. The *ost1* and OE-*OST1* plants crossed with OE-*CAP1* plants expressing *Lifeact-GFP.* Rosette leaves from 3 to 4-week-old plants were incubated in opening buffer for 3 h at 24°C. And then leaves were transferred to stomatal closure buffer containing ABA, Jasp or LatA. Then leaves were cut and observed with Olympus spinning disk confocal microscope using a × 60 oil immersion objective. The image of a single guard cell was obtained by Photoshop CS6. And the LPX plug-in of ImageJ was used to skeletonize the image and analyze skewness and average fluorescence density of actin filaments. GraphPad Prism 8 was used for graphs.

### Protein purification

The vectors of GST (*p*GEX-6P) or His (*p*ET28a) tag fusion proteins were transformed into *Escherichia coli* strain Rosetta. Protein was expressed under induction of 0.5 mM isopropyl β-D-thiogalactoside (IPTG) for 16 h at 16°C. Ni-NTA agarose or glutathione-sepharose beads were used to purify fusion proteins.

### In vitro GST pull down

GST-RCAR12 or GST (5 μg) were incubated with His-CAP1 (5 μg) and 25 μL glutathione S-Sepharose 4B (GS4B, GE Healthcare, PA, USA) resin in 500 μL binding buffer containing 25 mM Tris, pH 7.5, 150 mM NaCl, 1% Triton X-100 and ABA. After incubation for 2 h at 4°C, the mix was washed five times with binding buffer and resuspended with 2 X SDS-PAGE buffer. The pulled-down proteins were analyzed by immunoblotting using anti-GST and anti-His antibodies (Transgen, Beijing, China).

### RT-qPCR analysis

Total RNA was extracted from plants by RNAiso Plus (Takara). The Online tool OligoArchitect was used to design quantitative primers. Using the 2^-ΔΔCt^ to calculate relative differences. *Actin* in *Arabidopsis* or *EF1α* in *N. benthamiana* was used as internal control gene. At least three times were performed and three repeats each time. Primers for reverse transcription (RT)-qPCR were listed in S1 Table.

### Phosphorylation assay

An in vitro phosphorylation assay was performed using ATP-gamma-S as previously described [70]. CAP1 was incubated with OST1 or kinase-dead OST1^K50R^ in 50 μL kinase buffer containing 50 mM Tris-HCl (pH 7.5), 5 mM MgCl_2_, 1 mM DTT and 1 mM ATP-gamma-S. The mixture was incubated at 30°C for 1 h, and 2.5 mM p-Nitrobenzyl mesylate was added and incubated for another 2 h. The reaction was stopped with 2 X SDS loading buffer. The phosphorylated proteins were tested by immunoblotting using thiophosphate ester antibody and total protein inputs were assessed by Coomassie brilliant blue (CBB) staining.

For the in vivo phosphorylation assay, the total proteins from 10-d seedlings of OE-*CAP1* and *ost1*:OE-*CAP1* were extracted using lysis buffer (25 mM HEPES pH 7.5, 5 mM EDTA, 5 mM EGTA, 2 mM DTT, 1 mM Na_3_VO_4_, 5 mM NaF, 1 X cOmplete Protease Inhibitor Cocktail). The mixture was centrifuged twice at 12 000 rpm at 4°C for 20 min. The supernatant was separated using a phos-tag gel and detected with Flag antibody.

### Phosphorylation site identification

To identify the specific amino acids of CAP1 phosphorylated by OST1, recombinant protein GST-OST1 was mixed with His-CAP1 in a total volume of 50 µL reaction buffer containing 25 mM Tris-HCl (pH 7.5), 10 mM MgCl_2_, 1 mM KCl, and 1 mM ATP. The reaction mixture was incubated at 30°C for 1 h before adding SDS-loading buffer. After SDS-PAGE, His-CAP1 protein glue strips were extracted for mass spectrometry analysis.

The mass spectrometry analysis in vivo was performed by the Bioprofile Company (Shanghai, China). Gels containing immunoprecipitated CAP1–6Flag were dissolved in lysis buffer with acetonitrile, TCEP and chloroacetic acid (ACN). Ammonium bicarbonate (NH_4_HCO_3_) and trypsin buffer were added to each sample and the samples incubated overnight at 37°C. ACN and TFA (trifluoroacetic acid) were added and then the solution was subjected to ultrasonication. After digestion, chromatographic separation was performed using an Easy-nLC1200 chromatographic system (Thermo Scientific). The peptides were analyzed using the Q-Exactive HF-X mass spectrometer (Thermo Scientific).

### Quantification and statistical analysis

The gray value of band from immunoblots, stomatal aperture, and the density and skewness of actin filaments were measured by ImageJ. Statistical analysis was examined using GraphPad Prism 8.

## Supporting information

S1 Fig Analysis of the interactions between CAP1 and RCARs. (A) Yeast-two-hybrid assay. The serial dilutions (1, 0.1, 0.01, 0.001) were spotted on selective dropout (L, Leucine; W, Tryptophane; H, Histidine; A, Adenine) medium. BD (DNA-binding domain) indicates the *p*GBKT7 vector; AD (transcription activation domain) indicates the *p*GADT7 vector. (B) BiFC assay in *N. benthamiana* leaf epidermal cells. Scale bars = 50 μm. The images are the representative of three independent experiments (*n* = 3). (C) GST pull-down assay. His-CAP1 was incubated with GST-RCARs or GST and detected by immunoprecipitation using anti-GST and anti-His antibodies. GST served as a negative control. The blots shown are representative for two experiments. The images are the representative of three independent experiments (*n* = 2).
(TIF)

S2 Fig Expression pattern of RCAR12. The fluorescence of RCAR12-GFP was observed in guard cells. Scale bar = 20 μm.
(TIF)

S3 Fig Analysis of the binding domain of CAP1 by Y2H. Yeast-two-hybrid interactions among CAP1 with its variants by spotting serial dilutions (1:10, 1:100, 1:1000) of yeast on selective dropout media. The images are the representative of three independent experiments (*n* = 3). (B) Co-IP results demonstrating the self-association CAP1 in the leaves of *N. benthamiana*. GFP-2Flag served as a negative control. The immunoblots shown are representative of two independent experiments (*n* = 2) with the same results.
(TIF)

S4 Fig The identification of transgenic plants. (A) The protein levels of CAP1 in complementation lines. Total protein from ten-day-old seedlings were extracted. (B) The expression of *CAP1* in Col-0, com-CAP1-GFP and OE-*CAP1*. Values are mean ± SD (*n* = 3). (C) The GFP signals in complementation genetic line. Scale bar = 10 μm. (D) The co-localization of CAP1 with F-actin. Protoplasts from the com-CAP1-GFP seedlings were extracted and then stained with phalloidin-405. (E) The protein levels of CAP1 in overexpressing *CAP1* plants. Protein levels of CAP1 in OE-*CAP1* #23, OE-*CAP1* #18 and OE-*CAP1* #39 were tested by immunoblotting using anti-Flag antibody. The images are the representative of three independent experiments (*n* = 3).
(TIF)

S5 Fig The stomatal aperture in response to ABA. (A) ABA-induced stomatal closure in Col-0, *cap1–1* and complementation lines (com-CAP1 -GFP) plants. (B) ABA-induced stomatal closure in Col-0 and OE-*CAP1* plants. Mock and ABA: without and with the treatment of 20 μM ABA for 1 h. Scale bars = 20 μm.
(TIF)

S6 Fig Identification of plants. (A) The levels of F-actin labeling GFP in plants were tested with anti-GFP antibody. The loading of proteins was staining with Coomassie Brilliant Blue (CBB). The images are the representative of three independent experiments (*n* = 3). (B) The phenotype of drought tolerance in Col-0, *cap1–1* and OE-*CAP1* #23 labeling F-actin in (A). Three-week-old seedlings were subjected to drought stress by withholding water for 15-day and then rewatered for recovery. The numbers indicate the survival rate. Values are mean ± SD.
(TIF)

S7 Fig The analysis of qRT-PCR and protein levels in Fig 4A. (A) *Elongation factor 1α* (*EF1α*) was used as an internal control. nLUC or cLUC indicates N- or C-terminal of LUC. (B) Immunoblot confirmed the protein expression of nLuc and cLuc fusions, as determined with anti-nLuc and anti-cLuc antibodies.
(TIF)

S8 Fig RCAR12 inhibits the F-actin-binding activity of CAP1. (A) CAP1 was co-pelleted with F-actin in pellet (P), while BSA (as a negative control) in supernatants (S). CAP1 (3 μM), BSA (3 μM), F-actin (3 μM) were used. (B) The high-speed F-actin cosedimentation assays. Supernatants (S) and pellet (P) fractions of CAP1 (3 μM) were co-pelleted with RCAR12 (3 μM), F-actin (3 μM) and ABA (1 μM), respectively. The images are the representative of three independent experiments (A and B, *n* = 3). (C) Quantification of the amount of CAP1 in the supernatant in (B) using ImageJ. Data are presented as mean ± SD from three independent experiments (*n* = 3). The results were analyzed with Student’s *t*-test. ^**^P < 0.01. ns, no significance.
(TIF)

S9 Fig Interaction of CAP1 with ADF4. (A) BiFC assay. Each pair of constructs were transiently co-expressed in *N. benthamiana* leaf epidermal cells and analyzed by confocal microscopy after 96 h agro-infiltration. Scale bars = 50 μm. The images are the representative of three independent experiments (*n* = 3). (B) Depolymerization of 3 µM F-actin (10% pyrene labeled) in the presence of CAP1 (3 μM), RCAR12 (2 μM) or ADF4 (3 μM). F-actin depolymerization was monitored by tracking the decrease in pyrene fluorescence per minute. a.u., arbitrary units. Values indicate mean ± SD (*n* = 3).
(TIF)

S10 Fig Identification of RCAR12-binding site of CAP1. (A) Luciferase complementation imaging (LUI) assay. The indicated constructs were transiently co-expressed in *N. benthamiana* leaves. Leaves were photographed after 3-day of cultivation. The images are the representative of three independent experiments (*n* = 3). (B) The analysis of qRT-PCR. *Elongation factor 1α* (*EF1α*) was used as an internal control. (C) Representative confocal images of F-actin stained with Alexa488-Phalloidin. CAP1^M319^ (3 μM), ADF4 (3 μM), RCAR12 (1 μM). Scale bar = 5 μm. (D) Quantification of the length of F-actin in (C) by ImageJ. More than 100 filaments were measured for each treatment. Values are mean ± SD, *n* = 3 biological repeats. *P* < 0.05, as determined by one-way ANOVA with Tukey’s test.
(TIF)

S11 Fig The identification of plants. Proteins were extracted from 10-d seedlings, then analyzed by Western blot with anti-Flag, anti-MYC, and anti-GFP antibodies. The loading of proteins was staining with Coomassie Brilliant Blue (CBB). The images are the representative of three independent experiments (*n* = 3).
(TIF)

S12 Fig Identification of the phosphorylation sites of CAP1 in Arabidopsis. (A) Relative phosphopeptide signals of the peptides containing phosphorylated phosphorylated Ser246/249/250/253–256 residues in OE-*CAP1* and *ost1*:OE-*CAP1* seedlings based on LC–MS data. Values are the means ± SD of two biological replicates. Asterisk indicates statistically significant differences (*P < 0.05, Student’s t test, two-sided). (B) Interaction between OST1 and CAP1 validated by BiFC assays. The images are the representative of three independent experiments (*n* = 3). NLS-mCherry indicates nuclear localization. Immunoblot was used to test YFP and OST1 protein levels with anti-GFP and anti-MYC antibodies. We repeated the experiment four times with similar results and at least 8 leaves were observed per time. Scale bar = 50 μm.
(TIF)

S13 Fig OST1 regulates the F-actin in guard cells. (A) Representative images of actin networks before (CK) and after 200 nM LatA treatment. Scale bars = 10 µm. (B) Statistical analysis of filament average fluorescence intensity of guard cells under the normal condition (CK) or LatA treatment. Filaments in more than 20 guard cells were analyzed per genotype. Values are mean ± SEM from three independent experiments (*n* = 3). ** *P* < 0.05 based on one-way ANOVA with Tukey’s test.
(TIF)

S1 Video The F-actin dynamics in guard cell from Col-0. (AVI)

S2 Video The F-actin dynamics in guard cell from the *cap1–1* mutant. (AVI)

S3 Video The F-actin dynamics in guard cell from OE-CAP1 #23 plants. (AVI)

S1 Table Primers used in this study. (XLSX)

S2 Table Data shown in this study. (XLSX)

S3 Table Data shown in this study. (XLSX)

S4 Table Data shown in this study. (XLSX)

S5 Table Data shown in this study. (XLSX)

S6 Table Data shown in this study. (XLSX)

S7 Table Data shown in this study. (XLSX)

S8 Table Data shown in this study. (XLSX)

S9 Table Data shown in this study. (XLSX)

S10 Table Data shown in this study. (XLSX)

S11 Table Data shown in this study. (XLSX)

S12 Table Data shown in this study. (XLSX)

S13 Table Data shown in this study. (XLSX)

S14 Table Data shown in this study. (XLSX)

S15 Table Data shown in this study. (XLSX)

S16 Table Data shown in this study. (XLSX)

S17 Table Data shown in this study. (XLSX)

S18 Table Data shown in this study. (XLSX)

S19 Table Data shown in this study. (XLSX)

S20 Table Data shown in this study. (XLSX)

S21 Table Data shown in this study. (XLSX)

S22 Table Data shown in this study. (XLSX)

S23 Table Data shown in this study. (XLSX)
