# Supplementary figures and images for: The RCAR12-CAP1-OST1 module controls ABA-mediated stomatal closure in Arabidopsis
Source: PLoS Genet. 2026 Apr 28;22(4):e1012092. doi: 10.1371/journal.pgen.1012092 (PMC13123957; doi:10.1371/journal.pgen.1012092)

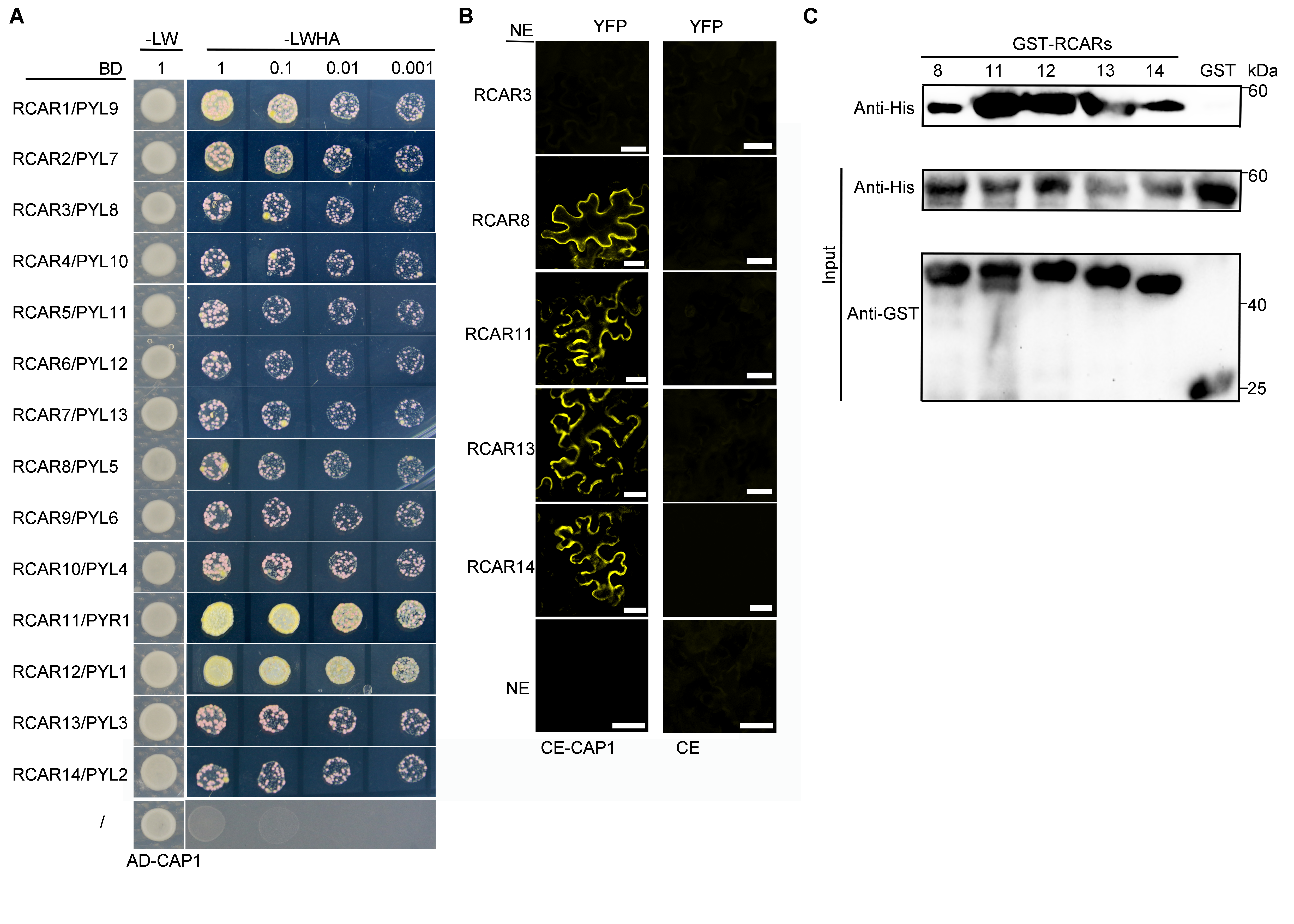

Supplement: S1 Fig — (A) Yeast-two-hybrid assay. The serial dilutions (1, 0.1, 0.01, 0.001) were spotted on selective dropout (L, Leucine; W, Tryptophane; H, Histidine; A, Adenine) medium. BD (DNA-binding domain) indicates the pGBKT7 vector; AD (transcription activation domain) indicates the pGADT7 vector. (B) BiFC assay in N. benthamiana leaf epidermal cells. Scale bars = 50 μm. The images are the representative of three independent experiments (n = 3). (C) GST pull-down assay. His-CAP1 was incubated with GST-RCARs or GST and detected by immunoprecipitation using anti-GST and anti-His antibodies. GST served as a negative control. The blots shown are representative for two experiments. The images are the representative of three independent experiments (n = 2). (TIF) [file pgen.1012092.s001.tif]

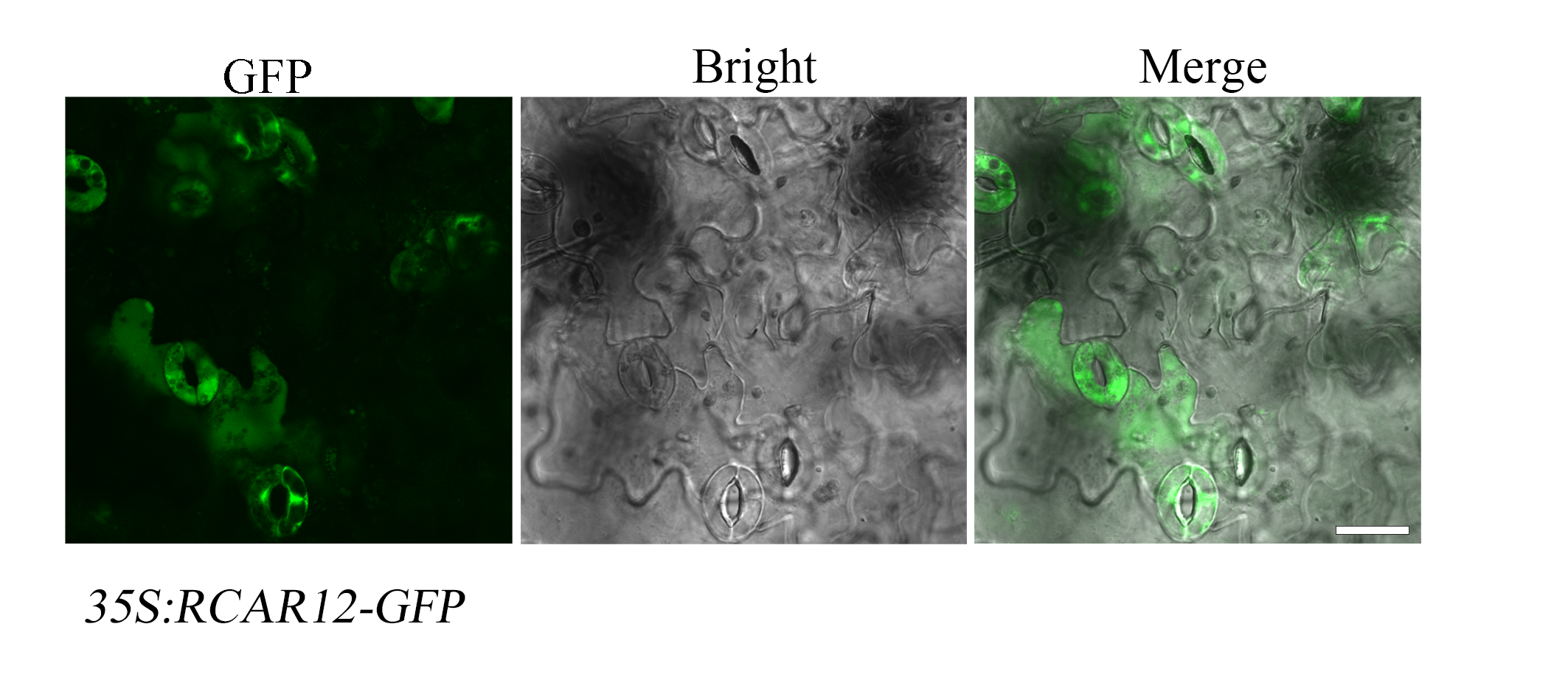

Supplement: S2 Fig — The fluorescence of RCAR12-GFP was observed in guard cells. Scale bar = 20 μm. (TIF) [file pgen.1012092.s002.tif]

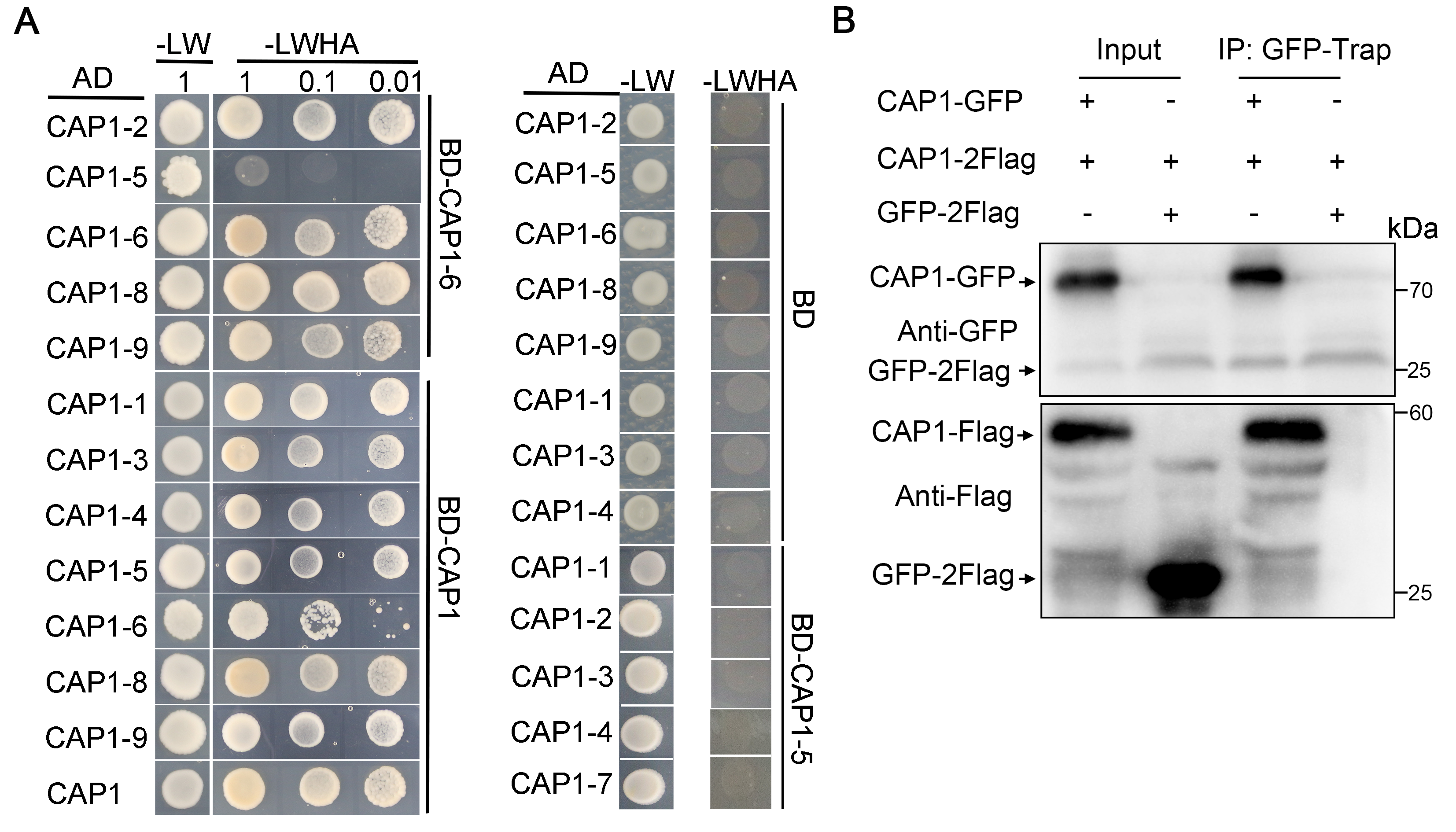

Supplement: S3 Fig — Yeast-two-hybrid interactions among CAP1 with its variants by spotting serial dilutions (1:10, 1:100, 1:1000) of yeast on selective dropout media. The images are the representative of three independent experiments (n = 3). (B) Co-IP results demonstrating the self-association CAP1 in the leaves of N. benthamiana. GFP-2Flag served as a negative control. The immunoblots shown are representative of two independent experiments (n = 2) with the same results. (TIF) [file pgen.1012092.s003.tif]

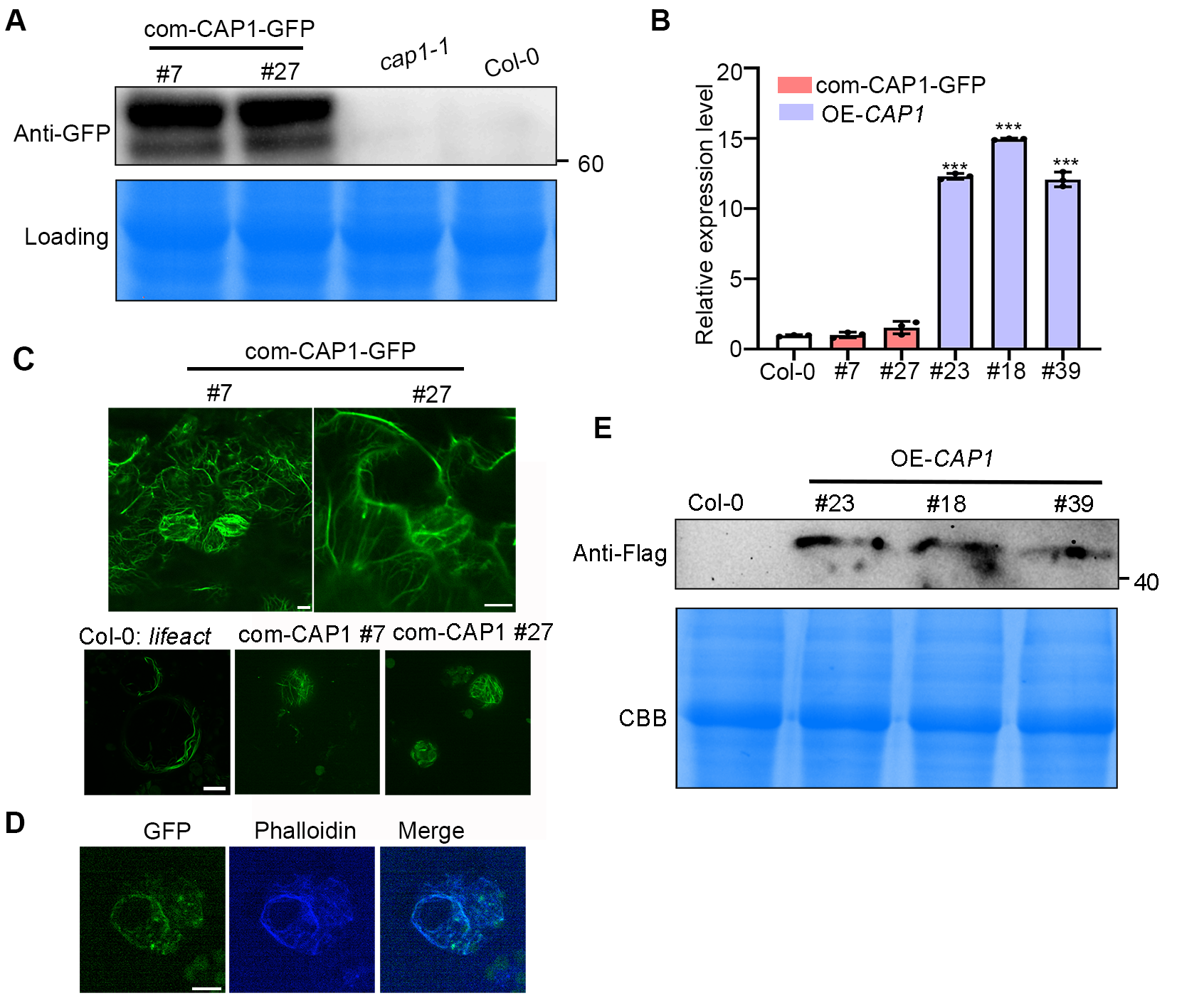

Supplement: S4 Fig — (A) The protein levels of CAP1 in complementation lines. Total protein from ten-day-old seedlings were extracted. (B) The expression of CAP1 in Col-0, com-CAP1-GFP and OE-CAP1. Values are mean ± SD (n = 3). (C) The GFP signals in complementation genetic line. Scale bar = 10 μm. (D) The co-localization of CAP1 with F-actin. Protoplasts from the com-CAP1-GFP seedlings were extracted and then stained with phalloidin-405. (E) The protein levels of CAP1 in overexpressing CAP1 plants. Protein levels of CAP1 in OE-CAP1 #23, OE-CAP1 #18 and OE-CAP1 #39 were tested by immunoblotting using anti-Flag antibody. The images are the representative of three independent experiments (n = 3). (TIF) [file pgen.1012092.s004.tif]

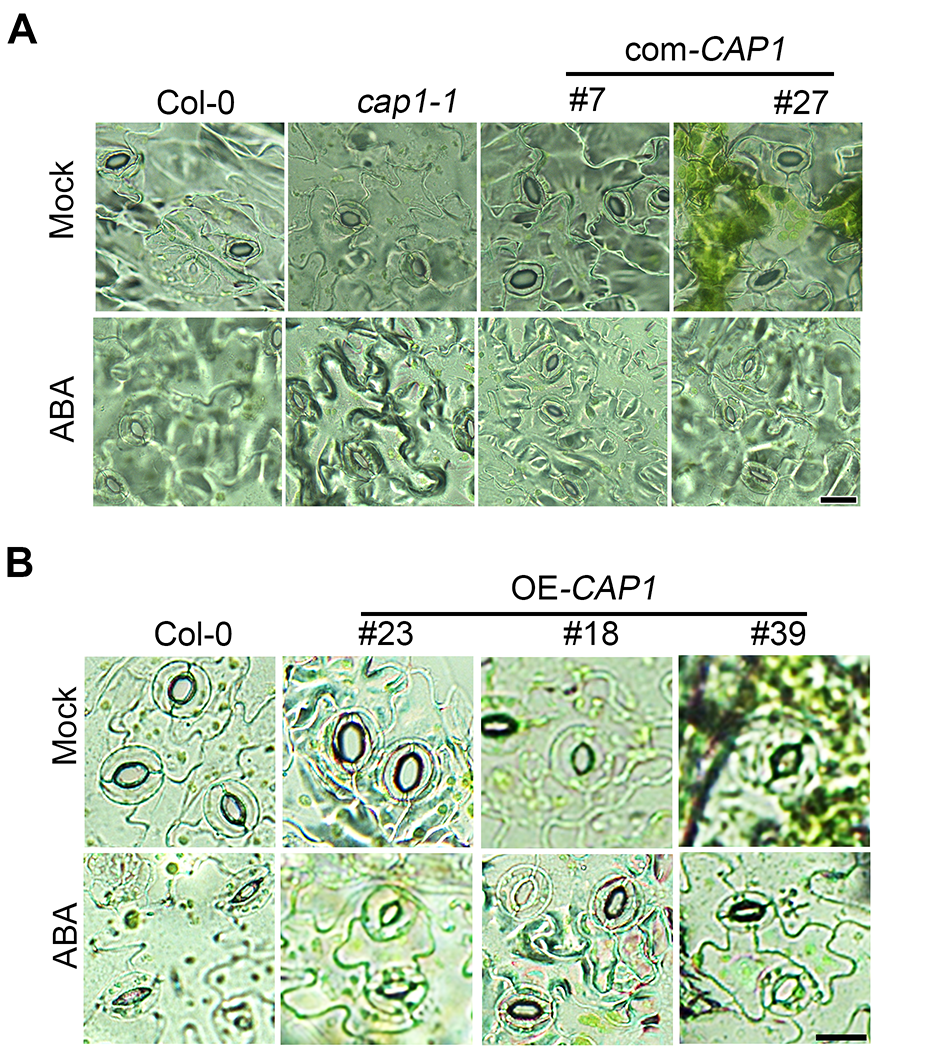

Supplement: S5 Fig — (A) ABA-induced stomatal closure in Col-0, cap1–1 and complementation lines (com-CAP1 -GFP) plants. (B) ABA-induced stomatal closure in Col-0 and OE-CAP1 plants. Mock and ABA: without and with the treatment of 20 μM ABA for 1 h. Scale bars = 20 μm. (TIF) [file pgen.1012092.s005.tif]

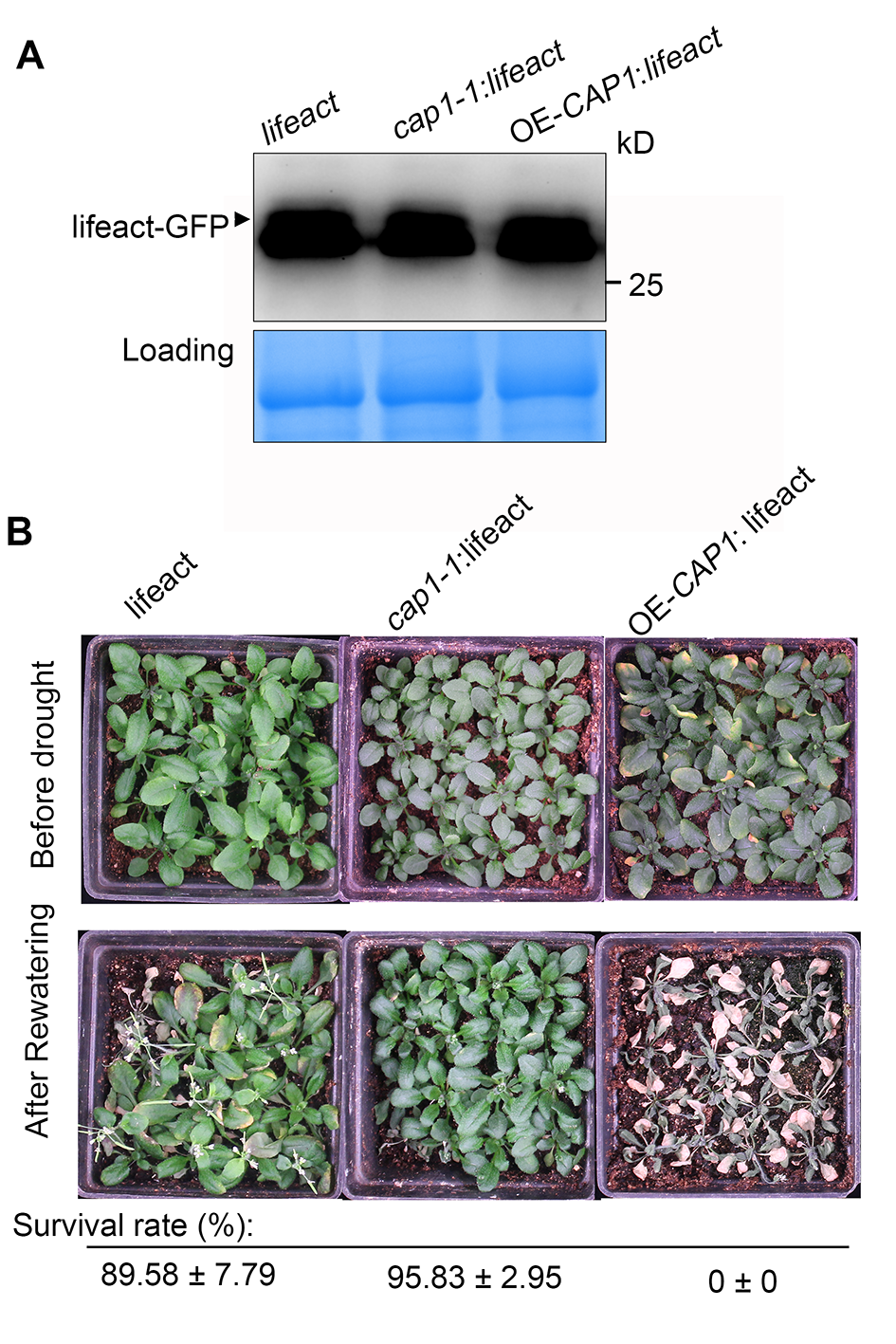

Supplement: S6 Fig — (A) The levels of F-actin labeling GFP in plants were tested with anti-GFP antibody. The loading of proteins was staining with Coomassie Brilliant Blue (CBB). The images are the representative of three independent experiments (n = 3). (B) The phenotype of drought tolerance in Col-0, cap1–1 and OE-CAP1 #23 labeling F-actin in (A). Three-week-old seedlings were subjected to drought stress by withholding water for 15-day and then rewatered for recovery. The numbers indicate the survival rate. Values are mean ± SD. (TIF) [file pgen.1012092.s006.tif]

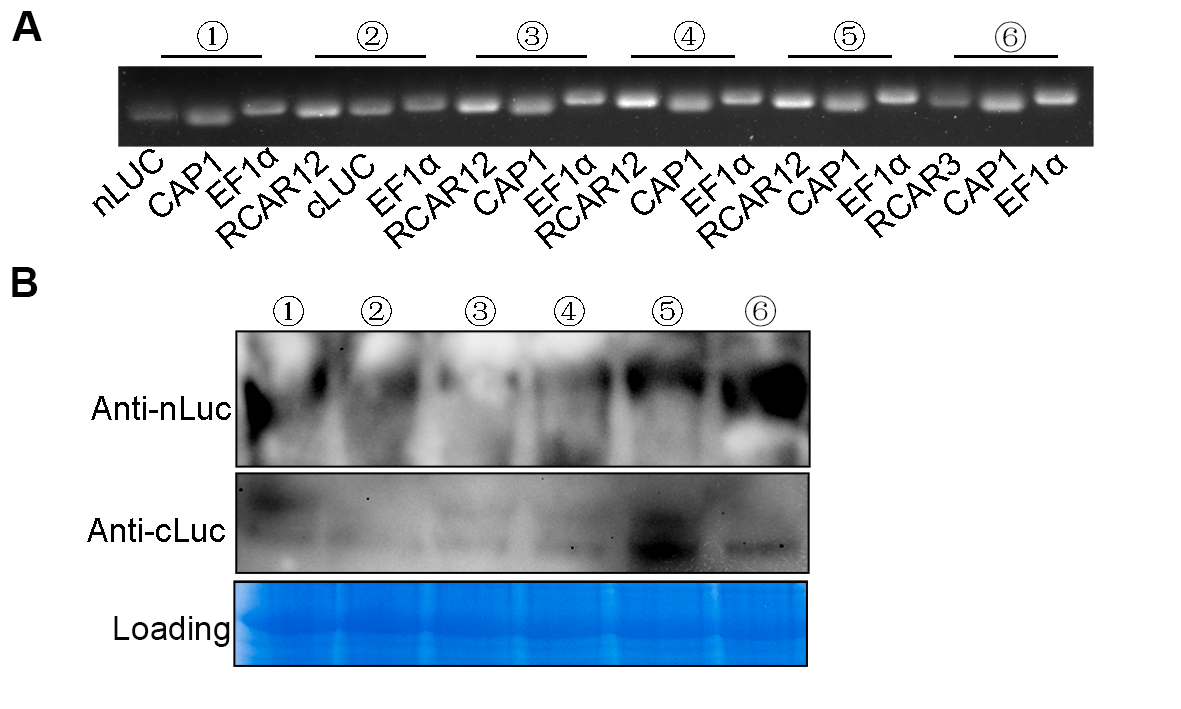

Supplement: S7 Fig — (A) Elongation factor 1α (EF1α) was used as an internal control. nLUC or cLUC indicates N- or C-terminal of LUC. (B) Immunoblot confirmed the protein expression of nLuc and cLuc fusions, as determined with anti-nLuc and anti-cLuc antibodies. (TIF) [file pgen.1012092.s007.tif]

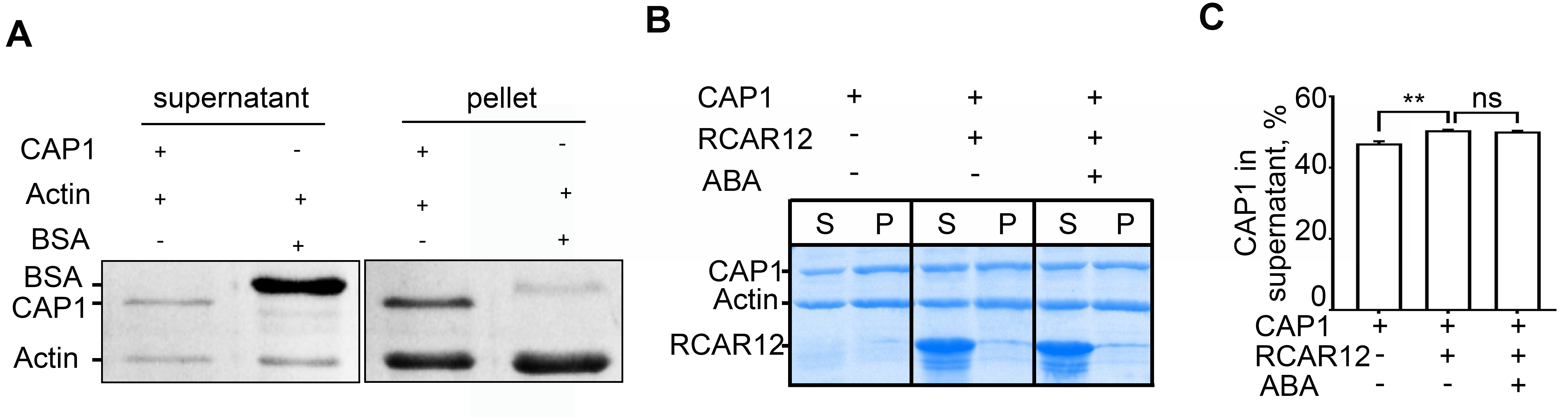

Supplement: S8 Fig — (A) CAP1 was co-pelleted with F-actin in pellet (P), while BSA (as a negative control) in supernatants (S). CAP1 (3 μM), BSA (3 μM), F-actin (3 μM) were used. (B) The high-speed F-actin cosedimentation assays. Supernatants (S) and pellet (P) fractions of CAP1 (3 μM) were co-pelleted with RCAR12 (3 μM), F-actin (3 μM) and ABA (1 μM), respectively. The images are the representative of three independent experiments (A and B, n = 3). (C) Quantification of the amount of CAP1 in the supernatant in (B) using ImageJ. Data are presented as mean ± SD from three independent experiments (n = 3). The results were analyzed with Student’s t-test. **P < 0.01. ns, no significance. (TIF) [file pgen.1012092.s008.tif]

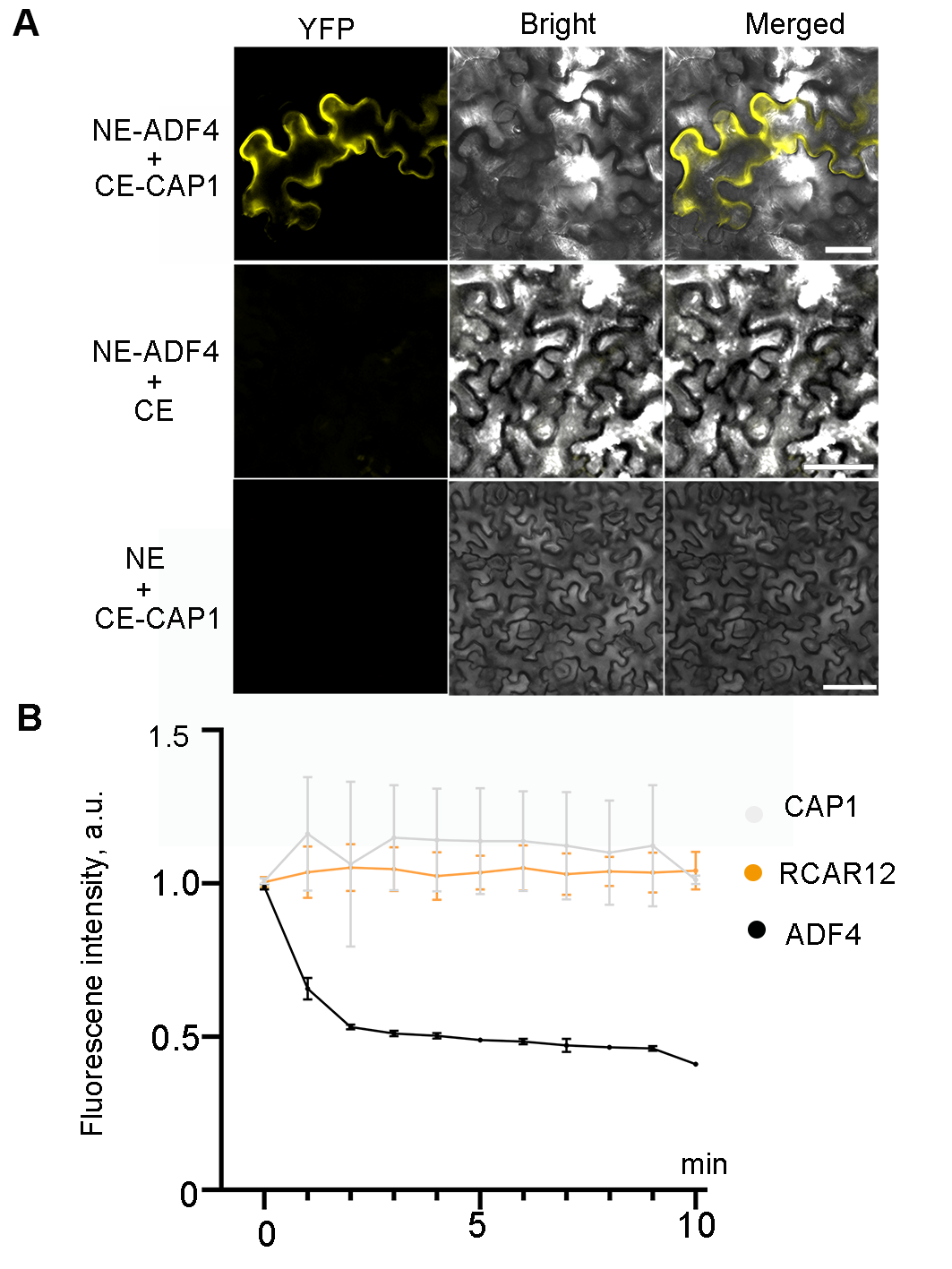

Supplement: S9 Fig — (A) BiFC assay. Each pair of constructs were transiently co-expressed in N. benthamiana leaf epidermal cells and analyzed by confocal microscopy after 96 h agro-infiltration. Scale bars = 50 μm. The images are the representative of three independent experiments (n = 3). (B) Depolymerization of 3 µM F-actin (10% pyrene labeled) in the presence of CAP1 (3 μM), RCAR12 (2 μM) or ADF4 (3 μM). F-actin depolymerization was monitored by tracking the decrease in pyrene fluorescence per minute. a.u., arbitrary units. Values indicate mean ± SD (n = 3). (TIF) [file pgen.1012092.s009.tif]

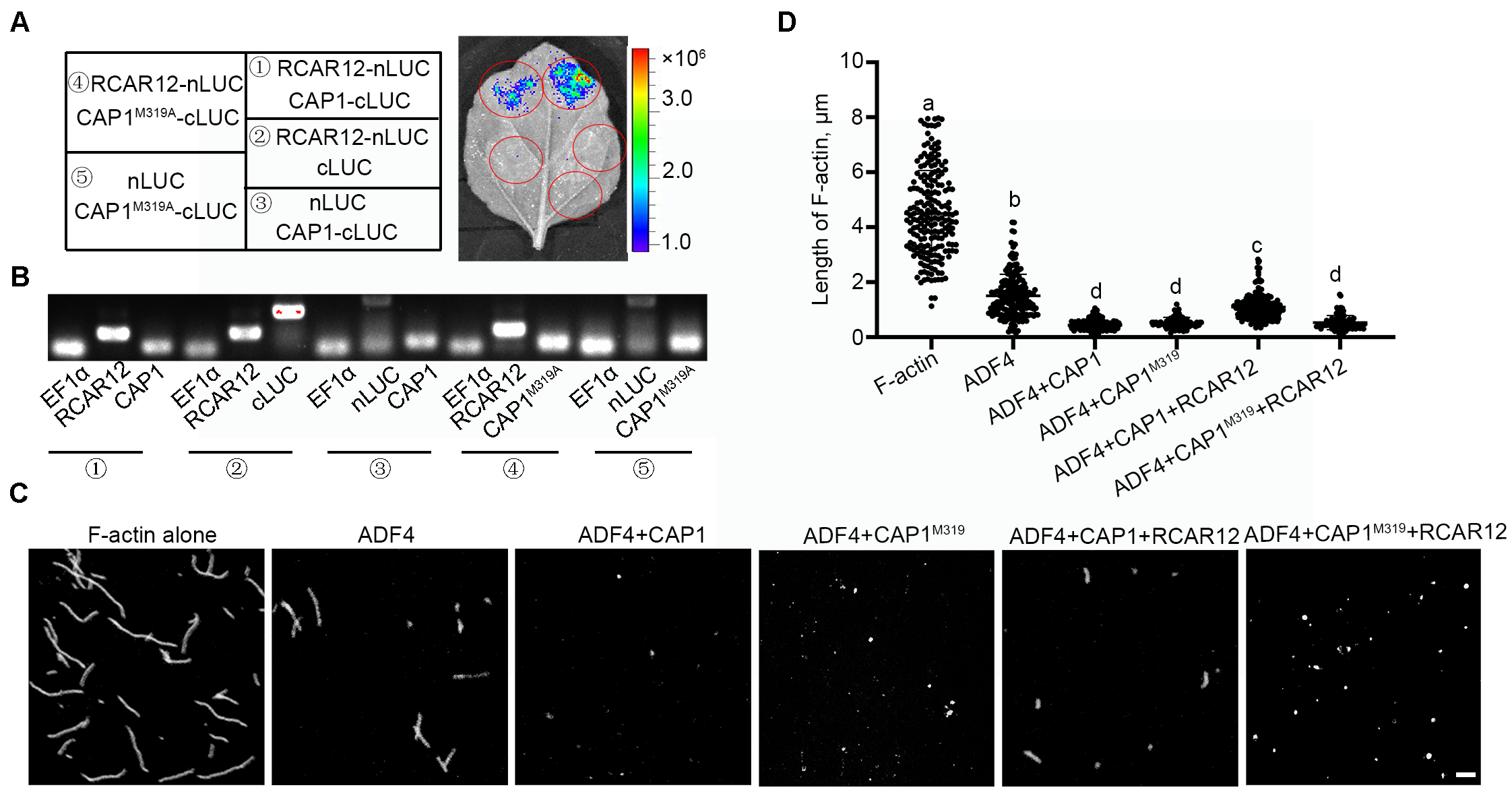

Supplement: S10 Fig — (A) Luciferase complementation imaging (LUI) assay. The indicated constructs were transiently co-expressed in N. benthamiana leaves. Leaves were photographed after 3-day of cultivation. The images are the representative of three independent experiments (n = 3). (B) The analysis of qRT-PCR. Elongation factor 1α (EF1α) was used as an internal control. (C) Representative confocal images of F-actin stained with Alexa488-Phalloidin. CAP1M319 (3 μM), ADF4 (3 μM), RCAR12 (1 μM). Scale bar = 5 μm. (D) Quantification of the length of F-actin in (C) by ImageJ. More than 100 filaments were measured for each treatment. Values are mean ± SD, n = 3 biological repeats. P < 0.05, as determined by one-way ANOVA with Tukey’s test. (TIF) [file pgen.1012092.s010.tif]

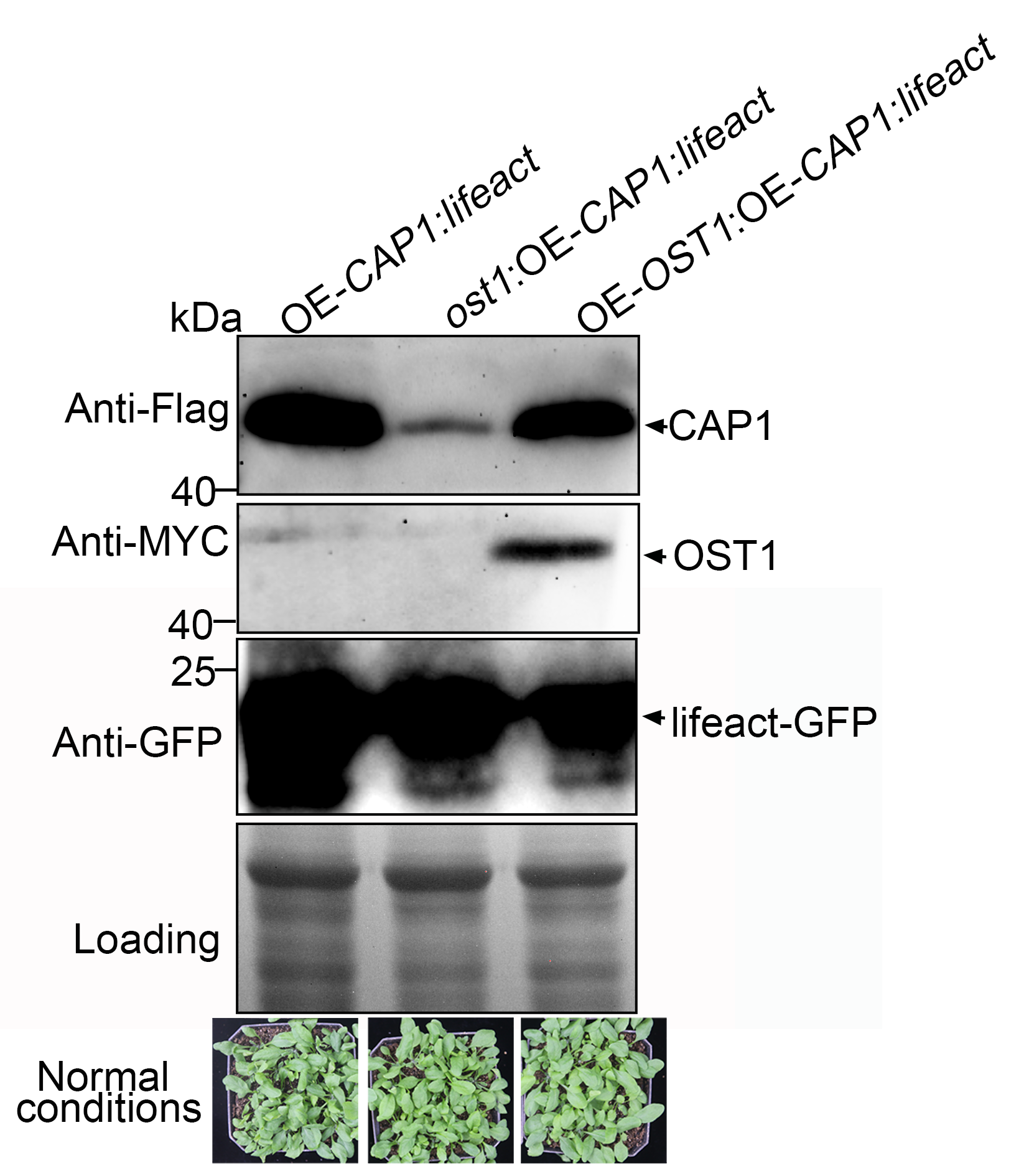

Supplement: S11 Fig — Proteins were extracted from 10-d seedlings, then analyzed by Western blot with anti-Flag, anti-MYC, and anti-GFP antibodies. The loading of proteins was staining with Coomassie Brilliant Blue (CBB). The images are the representative of three independent experiments (n = 3). (TIF) [file pgen.1012092.s011.tif]

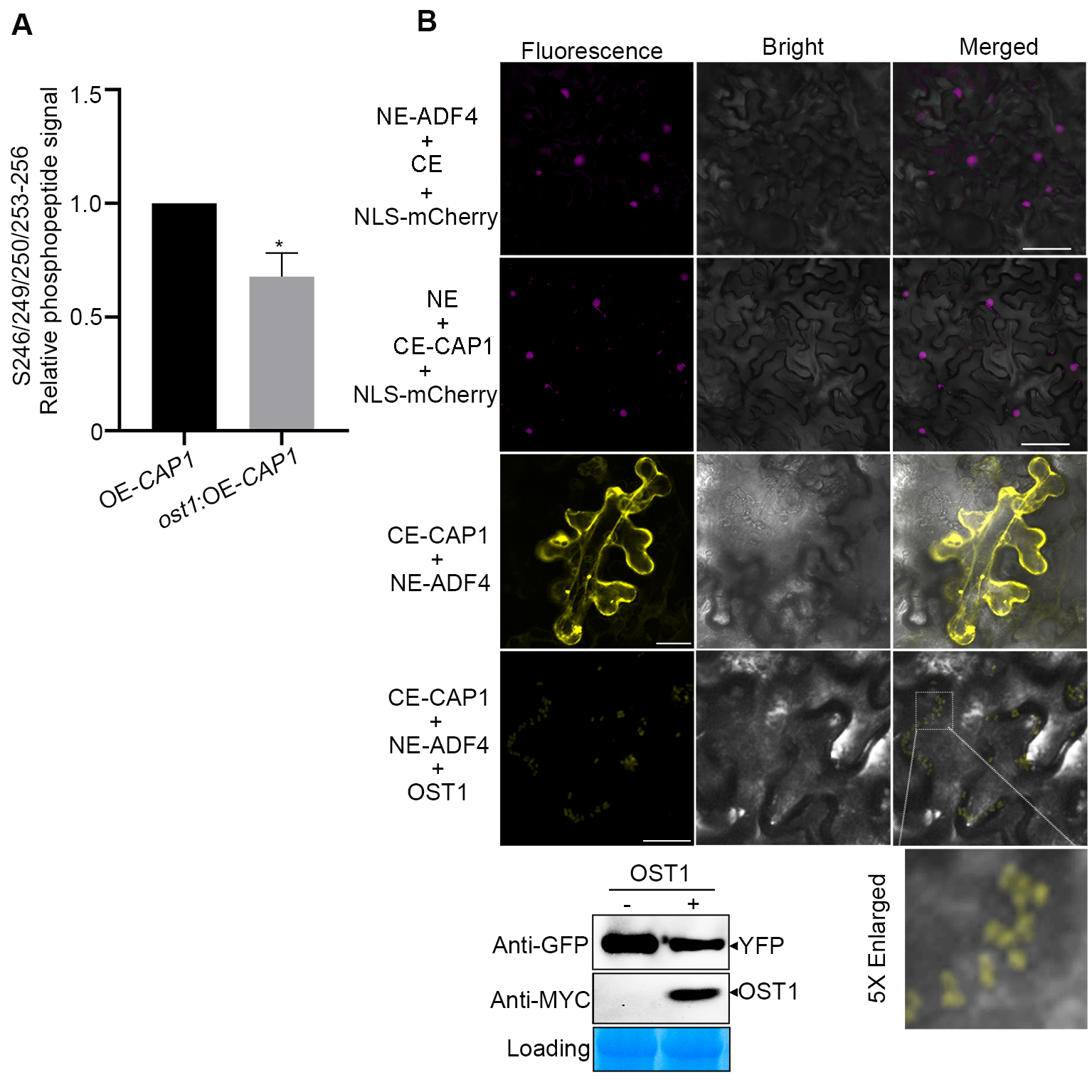

Supplement: S12 Fig — (A) Relative phosphopeptide signals of the peptides containing phosphorylated phosphorylated Ser246/249/250/253–256 residues in OE-CAP1 and ost1:OE-CAP1 seedlings based on LC–MS data. Values are the means ± SD of two biological replicates. Asterisk indicates statistically significant differences (*P < 0.05, Student’s t test, two-sided). (B) Interaction between OST1 and CAP1 validated by BiFC assays. The images are the representative of three independent experiments (n = 3). NLS-mCherry indicates nuclear localization. Immunoblot was used to test YFP and OST1 protein levels with anti-GFP and anti-MYC antibodies. We repeated the experiment four times with similar results and at least 8 leaves were observed per time. Scale bar = 50 μm. (TIF) [file pgen.1012092.s012.tif]

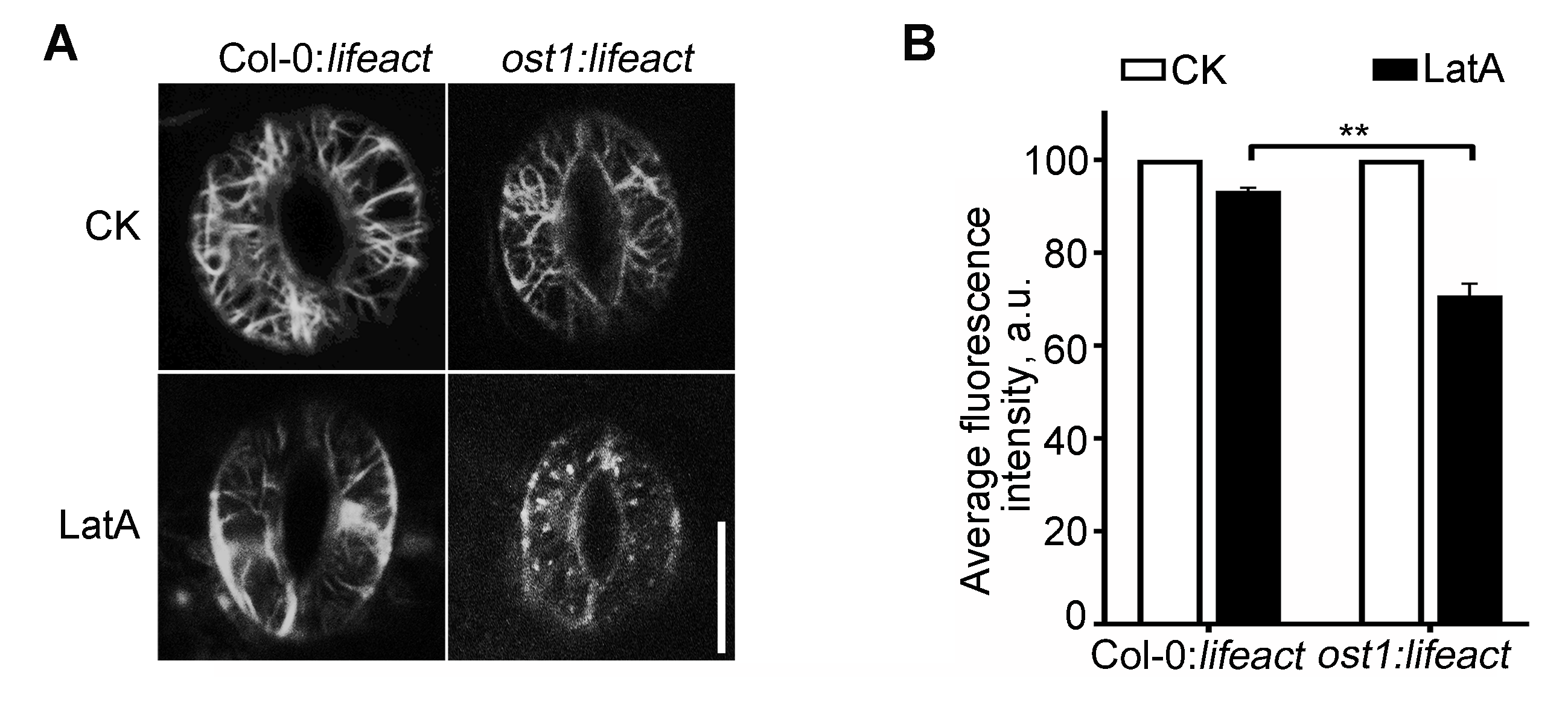

Supplement: S13 Fig — (A) Representative images of actin networks before (CK) and after 200 nM LatA treatment. Scale bars = 10 µm. (B) Statistical analysis of filament average fluorescence intensity of guard cells under the normal condition (CK) or LatA treatment. Filaments in more than 20 guard cells were analyzed per genotype. Values are mean ± SEM from three independent experiments (n = 3). ** P < 0.05 based on one-way ANOVA with Tukey’s test. (TIF) [file pgen.1012092.s013.tif]
